# Methodologies for population surveys on violence in rural areas: a scoping review

**DOI:** 10.1590/1980-220X-REEUSP-2025-0239en

**Published:** 2026-02-27

**Authors:** Carmem Layana Bandeira Bervig, Marta Cocco, Jaqueline Arboit, Ethel Bastos da Silva, Cristiane Cardoso de Paula, Manuel Gaspar da Silva Lisboa

**Affiliations:** 1 Universidade Federal do Rio Grande do Sul, Escola de Enfermagem, Porto Alegre, RS, Brazil.; 2 Universidade Federal de Santa Maria, Departamento de Ciências da Saúde, Palmeira das Missões, RS, Brazil.; 3 Universidade Federal de Santa Maria, Centro de Ciências da Saúde, Departamento de Enfermagem, Santa Maria, RS, Brazil.; 4 Universidade Nova de Lisboa, Faculdade de Ciências Sociais e Humanas, Departamento de Sociologia, Lisboa, Portugal.

**Keywords:** Violence, Rural Health, Surveys and Questionnaires, Scoping Review

## Abstract

**Objetive:**

To examine the methods used in population surveys concerning the epidemiology of violence against people living in rural areas.

**Method:**

Scoping review, conducted using the JBI method and reported according to PRISMA-ScR. The protocol was registered with OSF in August 2022 (DOI https://doi.org/10.17605/OSF.IO/WG6PU). The searches took place from July to September 2022, with an update on April 27, 2025, through keywords and controlled terms mapping, guided by the acronym Population, Concept and Context. The data sources included MEDLINE, Web of Science, LILACS, Scopus, EMBASE, CINAHL, Cochrane Library, grey literature, and unpublished studies. Two independent reviewers selected the studies.

**Results:**

One hundred and ten studies were included: 89.09% (n = 98) with a cross-sectional design; 46.36% (n = 51) with adults; and 56.36% (n = 62) with women. In 37.27% (n = 41) of the studies, the sampling technique was not described; 59.09% (n = 65) used questionnaires; and 69.09% (n = 76) performed descriptive and inferential statistical analysis.

**Conclusion:**

Heterogeneity was observed regarding the instruments, sampling, and methodological designs of the surveys.

## INTRODUCTION

Violence constitutes a serious global health problem that demands intersectoral efforts and individual and institutional resources, often limited for its effective tackling^(1)^. Statistically, this is a phenomenon that is difficult to measure, given its complexity, social invisibility, and underreporting^(1,2)^. The World Health Organization defines violence as the intentional use of force or power, that is real or a threaten, against oneself or others, that either results in or has a high likelihood of resulting in injury, death, psychological harm, developmental disability or deprivation^(3)^.

In rural areas, violence takes on even more complex dimensions due to geographical distance from cities, lack of public transportation, barriers to accessing healthcare services, poor infrastructure, and shortage of supplies^(4)^. People living in these areas face multiple forms of violence – physical, psychological, sexual, and financial – perpetrated by family members, colleagues, community members, and sometimes healthcare professionals^(3,4 5 6 7)^. Although it also occurs in urban areas, underreporting is particularly high in rural areas due to difficulties in accessing protective services and to sociocultural factors that normalize violence^(1)^. These obstacles increase the vulnerability of the rural population, reinforcing the invisibility of the phenomenon^(4)^.

Population surveys are essential tools for the epidemiological measurement of violence, allowing for the estimation of prevalence rates, mapping of associated factors, and support for public policies aimed at prevention and coping. These studies provide information on the types of violence, risk factors, consequences, and impacts in different social contexts, and are fundamental to understanding the scope of the problem^(8,9)^. Important national surveys, such as the National Health Survey (PNS)^(9)^, include rural areas in their sampling; however, they rarely explore the health outcomes and the particularities of data collection extensively in rural contexts regarding violence.

Despite the relevance of surveys, there is a lack of systematizations to comprehensively examine the methods used in population surveys aimed at measuring violence in rural areas. This gap compromises data comparability, limits the strengthening of the evidence base, and hinders the planning of intersectoral actions and the monitoring of equity policies. Population-based studies remain scattered, methodologically heterogeneous, and often inadequate to capture the territorial, cultural, and access- related uniqueness of the population residing outside urban centers. Thus, population surveys are central to filling this gap, as they collect information directly from residents, addressing different forms of violence^(9,10)^, revealing patterns of violence that are often overlooked in public policies and in the allocation of resources for the prevention and mitigation of the phenomenon^(8,9)^.

In light of the above, this study aims to guide the choice of more appropriate methods for conducting population surveys on violence in rural areas, focusing on data collection and analysis techniques that consider sociodemographic, cultural, economic variables and other social markers. Therefore, this scoping review aimed to examine the methods used in population surveys concerning the epidemiology of violence against people living in rural areas.

## METHOD

### Design of Study

This is a scoping review conducted in accordance with the Joanna Briggs Institute (JBI) method and reported according to the guidelines of *Preferred Reporting Items for Systematic reviews and Meta-Analyses extension for Scoping Reviews* (PRISMA-ScR)^(11,12)^. JBI is an international institution dedicated to the synthesis, transfer, and implementation of health evidence, recognized for the rigor and up-to-dateness of its designs for systematic and scoping reviews. Thus, the choice of this framework is justified by the methodological soundness it provides to the review^(11)^. It is important to highlight that the focus of this review is on the methodological strategies employed in population surveys, without delving into the categorization of the types of violence addressed, as this is a distinct analytical axis.

For its development, the following stages were taken: defining the objective, review question, and selection criteria; planning and executing the search strategy; selecting and extracting data; analyzing and presenting the data; and summarizing the evidence mapped in accordance with the review objective^(11)^.

Initially, the *Open Science Framework* (OSF), *Medical Literature Analysis and Retrieval System Online* (MEDLINE), *Cochrane Database of Systematic Reviews*, PROSPERO (*International prospective register of systematic reviews*) and JBI *Evidence Synthesis* were consulted*,* with no published or ongoing scoping reviews being identified regarding population-based surveys related to the epidemiology of violence in rural areas. Therefore, the review protocol was developed and publicly registered with OSF on August 20, 2022 (DOI https://doi.org/10.17605/OSF.IO/WG6PU).

### Identifying the Review Question

The review question was structured based on the acronym PCC (Population, Concept, and Context)^(11)^ with: P - people experiencing violence; C - population-based survey methods; and C - rural areas. Based on this mnemonic combination, the question was formulated: Which methods are used in population surveys concerning the epidemiology of violence against people living in rural areas?

### Selection Criteria

The inclusion and exclusion criteria were defined according to the elements of the PCC acronym. Population (P): studies with children, adolescents, adults and the older people in situations of violence, without any restriction regarding religion, sexual orientation, gender identity, disability or other social markers. The following age ranges were adopted: children (up to 12 years of age); adolescents (12 years of age completed to 18 years of age to be completed); adults (19 years of age completed to 59 years of age); and older people (60 years of age or older)^(5)^. Studies focusing on the health consequences of violence were excluded because they addressed clinical outcomes rather than methodological aspects, which does not correspond to the objective of this review. Concept (C): studies using population survey methods. Context (C): studies addressing the rural area. Studies analyzing data from urban and rural areas together were excluded.

Articles from experimental and quasi-experimental studies were included (randomized and non-randomized controlled clinical trials; before-and-after studies; time series); analytical observational studies (prospective and retrospective cohort studies, case-control studies, analytical cross-sectional studies); documentary research designs (such as medical record analysis); descriptive observational studies (case series, individual case reports, and descriptive cross-sectional studies); as well as grey literature (dissertations, theses, monographs); available in Portuguese, English, or Spanish. Qualitative studies, mixed methods studies, studies involving the creation or validation of instruments, intervention studies, preliminary notes, review studies, research protocols, and editorials were excluded. No restrictions were applied regarding the publication period.

### Search Strategies

The JBI recommends using a three-stage search strategy to retrieve eligible, published, and unpublished studies^(11)^. The bibliographic searches were carried out with the collaboration of a librarian specialized in health information retrieval and certified by JBI, who conducted the mapping of keywords and standardized terms in controlled vocabularies: Health Sciences Descriptors (DeCS), via the Regional Portal of the Virtual Health Library (VHL); Medical Subject Headings (MeSH) via PubMed; and Emtree (Embase subject headings), from the EMBASE database (Elsevier). In the first stage, an initial search was conducted combining keywords and descriptors in the titles of medical subjects in MEDLINE via PubMed and in the VHL Portal, to map the relevant terms. Then, the titles and abstracts of the retrieved articles were analyzed for commonly used keywords and index terms.

In the second stage, additional terms extracted from the titles, abstracts, and descriptors (MeSH/DeCS/Emtree) of the articles were included and applied to the title, abstract, and subject fields (Subject Headings, Mesh Terms, Emtree Terms, Keywords), allowing the formulation of a final search strategy, applied to PubMed in September 2022 (Chart 1). This strategy was later adapted to the other information sources included. The complete strategies for all sources and the extraction files are publicly available at *SciELO Data* (https://doi.org/10.48331/SCIELODATA.MU1AB9), ensuring transparency and reproducibility. In the third stage, the reference lists of the included studies were examined according to the eligibility criteria, to find other studies potentially relevant to the review.

**Chart 1:** PubMed search strategy applied on September 23, 2022 – Palmeira das Missões, RS, Brazil, 2023.

Search	Query	Results
#1	Search: “Violence”[mh] violence*[tiab] OR Crime*[tiab] OR Offense*[tiab] OR Abuse*[tiab] OR “Aggression”[mh] OR Aggression*[tiab] OR “Homicide”[mh] OR Homicide*[tiab] OR femicide*[tiab] OR mistreatment[tiab] Sort by: Most Recent	273,117
#2	Search: “Surveys and Questionnaires”[mh] OR Survey*[tiab] OR Nonrespondent*[tiab] OR Questionnaire*[tiab] OR Respondent*[tiab] OR “Health Surveys”[mh] OR “Health Survey”[tiab] OR “National Health Survey”[tiab] OR Prevalence[mh] OR Prevalence*[tiab] OR Incidence*[tiab] OR Epidemiology[mh] OR Epidemiolog*[tiab] OR “Health Surveys”[mh] OR Health Survey*[tiab] OR “population-based survey”[tiab] OR “Epidemiologic Studies”[mh] OR “Epidemiological Studies”[tiab] OR “Epidemiological Study”[tiab] OR “Epidemiologic Study”[tiab] OR “population-based household survey”[tiab] OR “population-based survey”[tiab] OR “population-based household survey”[tiab] OR “population survey”[tiab] Sort by: Most Recent	5,493,787
#3	Search: Rural Area*[tiab] OR Rural Zone*[tiab] OR “Rural Population”[mh] OR “Medium Communities”[tiab] OR Rural Communit*[tiab] OR Rural Population*[tiab] OR Rural Settlement*[tiab] OR Rural Spatial Distribution*[tiab] OR Small Communit*[tiab] OR “peasant farmers”[tiab] OR peasant[tiab] OR cottager*[tiab] OR Rural[tiab] OR countryside[tiab] OR “agricultural area”[tiab] OR “rural environment”[tiab] OR “rural land”[tiab] OR “rural dweller”[tiab] OR “rural people”[tiab] OR “rural resident”[tiab] OR “rural society”[tiab] OR “rural worker”[tiab] OR “village people”[tiab] OR “village population”[tiab] OR “village resident”[tiab] OR villager[tiab] Sort by: Most Recent	183,615
#4	Search: #1 AND #2 AND #3 Sort by: Most Recent	2,527

Source: Pubmed, (Org) author.

Data sources researched included: Medical Literature Analysis and Retrieval System Online (MEDLINE/PubMed), Web of Science Core Collection/Clarivate Analytics, Latin American and Caribbean Literature in Health Sciences (LILACS/VHL), SciVerse Scopus/Elsevier, EMBASE/Elsevier, Cumulative Index to Nursing and Allied Health (CINAHL/EBSCO), Cochrane Library/Wiley. The sources of unpublished studies and grey literature included: DART-E, EthoS, Open Access Theses and Dissertations (OATD), Open Gray, Networked Digital Library of Theses and Dissertations (NDLTD), ProQuest Dissertations & Theses Global (PQDT) and the Thesis Database of the Coordination for the Improvement of Higher Education Personnel (CAPES). The manual search for references was conducted using Google Scholar. The bibliographic searches were conducted from July to September 2022 and updated on April 27, 2025, reapplying the strategy adapted to each base.

### Selection of Sources of Evidence

After searching the data sources for the studies, all identified records were exported to the Endnote reference manager and, after removing duplicates, imported into *Rayyan software*. Next, two independent reviewers performed the screening by reading titles/abstracts in blind mode (*Blind ON/OFF)*. In cases where there was disagreement, a third reviewer was contacted.

The selection process was preceded by a pilot test of 25 titles/abstracts per reviewer. After team discussion and a consensus of 75% being reached, the selection process continued^(13)^. To ensure the reliability of the process, a checklist with eligibility criteria was developed. Conflicts between the reviewers were resolved by the third reviewer and, when necessary, contact was made via e-mail with the authors of the primary studies for clarification. In the second stage, the pre-selected studies were read in their entirety. The entire selection process was documented in a flowchart, in accordance with the guidelines of PRISMA-ScR^(12)^.

### Data Extraction

Data were extracted from the included articles by two reviewers independently and, in cases of disagreement, a third reviewer was consulted. A structured form was used^(11)^ in Microsoft Excel®, previously tested to verify its suitability for the review question. The reviewers were properly trained to conduct this stage. The information extracted included: author(s), year of publication, country of origin, objective, population and sample, study design, data collection and analysis techniques, as well as sociodemographic variables related to equity policies – disability, sexual orientation, gender identity, and other social markers of difference. The variable violence in rural areas was identified according to the definition adopted in each study, considering criteria such as self-declaration of rural residence, administrative classification of the territory, or geographic stratification used by the original authors.

### Data Analysis and Treatment

The collected evidence was analyzed descriptively and comparatively, and presented in tables and graphs. A narrative summary accompanies the results, relating the findings to the objective and review question.

### Ethical Aspects

Because this is a literature review, without direct involvement of human subjects and analyzing exclusively publicly available secondary data, this study did not require approval from a Research Ethics Committee. The protocol, however, was reviewed and registered in accordance with international best practice recommendations for systematic and scoping reviews. It is important to note that the copyright and integrity of the research analyzed were respected.

## RESULTS

**Figure 1:**
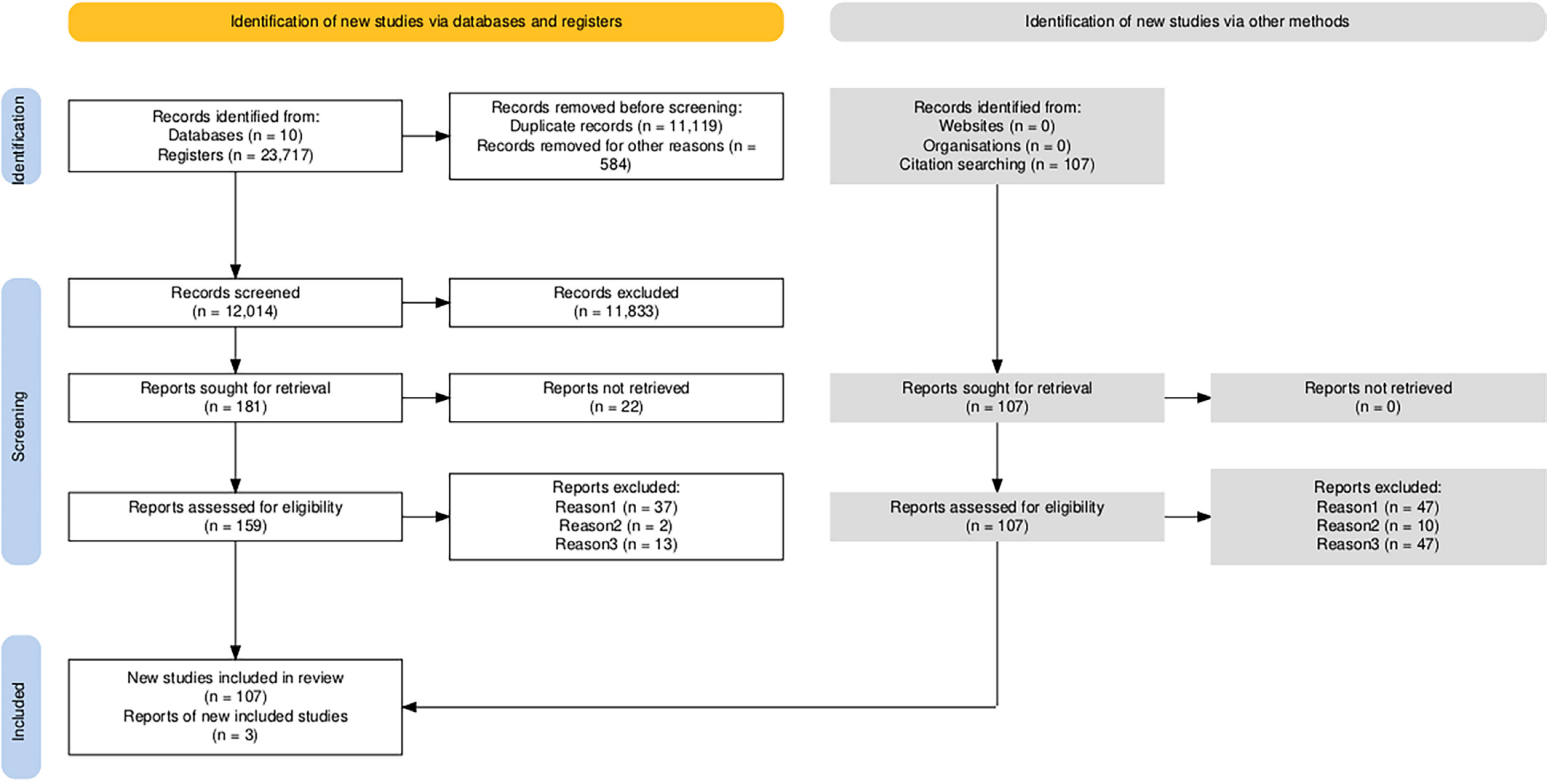
PRISMA 2020 flowchart for new systematic reviews, including searches in databases and registries – Palmeira das Missões/RS, Brazil, 2025.

Legend: *VHS (n = 542), Cochrane (n = 347), EBSCO – CINAHL and SocINDEX (n = 3601), Embase (n = 5075), ETHOS (n = 10), POPLINE (n = 99), PUBMED (n = 3089), PsycINFO (n = 200), Scopus (n = 6289), WOS (n = 4388).**Removed before selection – marked as ineligible. ***Excluded for not answering the review question. ****Reason 1: Population Reason 2: Concept Reason 3: Context.

The search resulted in the identification of 23,717 studies. After removing 11,119 duplicates and 584 ineligible records, 12,014 studies were submitted for screening by title and abstract. Of these, 11,833 were excluded for not answering the review question, and 181 were left for full reading. Based on the analysis of the reference lists, three additional studies were included. In the full reading, 22 were not retrieved and 49 were excluded for not meeting the inclusion criteria. Thus, 110 studies comprised the review analytical *corpus*, as demonstrated in the PRISMA flowchart (Figure 1).

**Figure 2:**
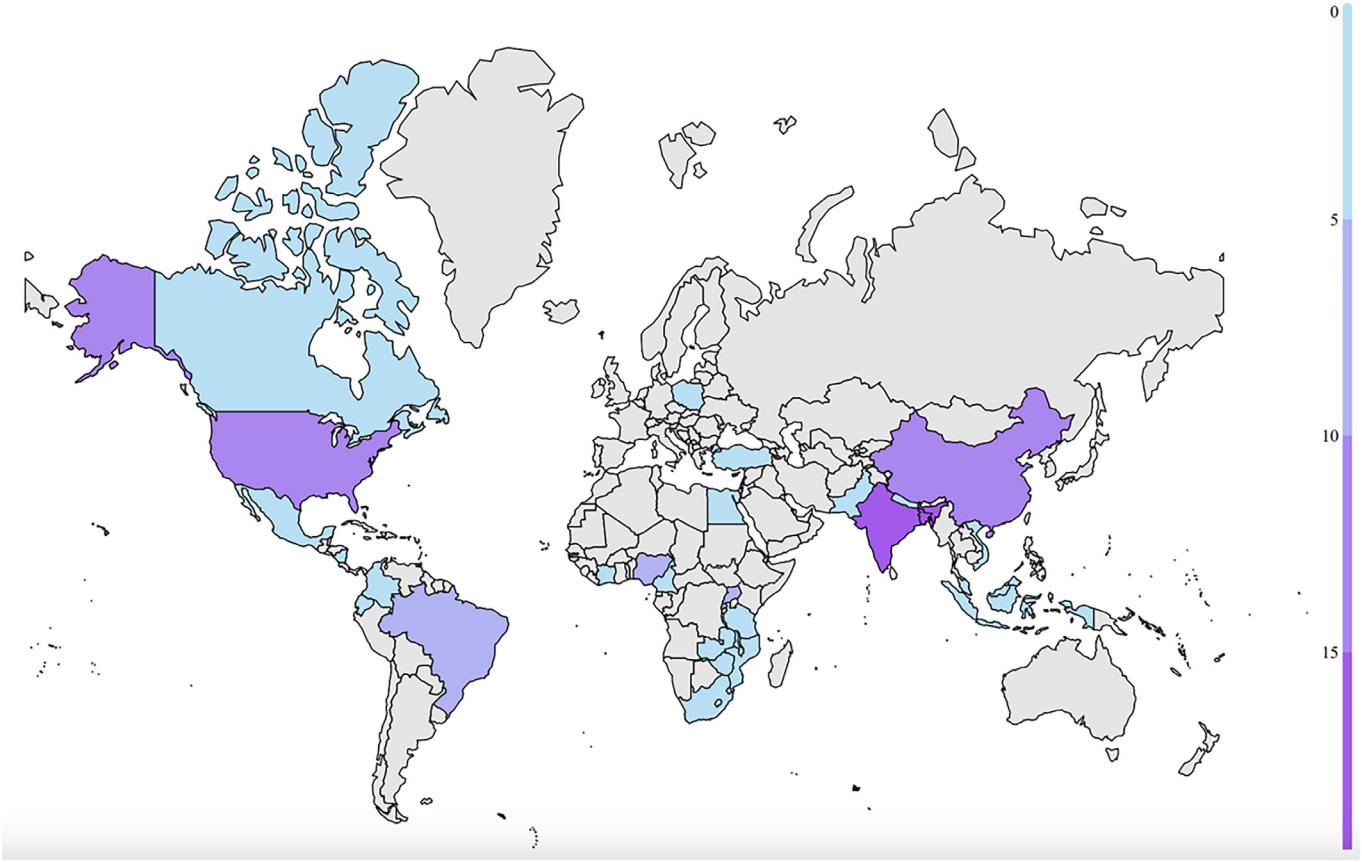
Geographic distribution of the studies included in the review – Palmeira das Missões/RS, 2025.

The geographical distribution of the included studies is presented in Figure 2, on a scale of 0 to 20. The map was created on the platform *Infogram* (free version). The more intense the color, the greater the number of studies per country. A predominance is observed in India (n = 20), Bangladesh (n = 15), the United States (n = 12), and China (n = 11).

**Figure 3:**
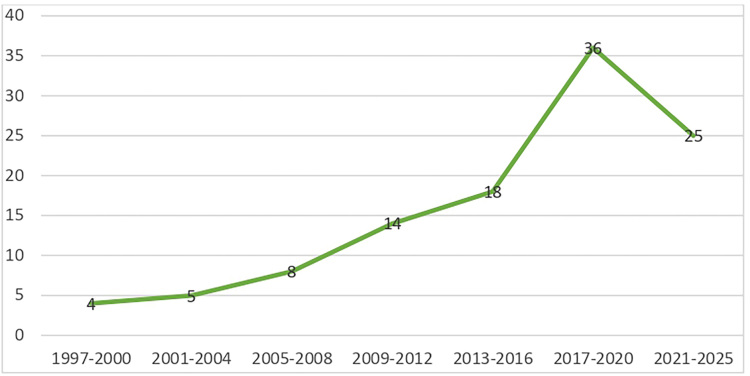
Number of studies by year of publication – Palmeira das Missões/RS, 2025.

Figure 3 shows the temporal distribution of studies published between 1997 and 2025, with the highest concentration from 2017 to 2020. Between 2020 and 2022, years marked by the COVID-19 pandemic, 28 studies were found.

All studies adopted a quantitative approach. Of these, 98 (89.09%) used a cross-sectional design; six (5.45%) were cohort studies; three (2.73%), case series; one (0.91%), a case-control; one (0.91%), a population-based survey; and one (0.91%), a household survey (Chart 2).

**Chart 2:** Chart with studies identification – Palmeira das Missões, RS, Brazil, 2025^(7,14–122)^.

Reference year	Design number of participants age range sex	Sampling technique	Data collection technique	Data collection local	Data analysis
2021^(7)^	Cross-sectional. 1,681 children of both sexes.	Multi-stage conglomerates	Interview	School	Inferential statistics
1997^(14)^	Cross-sectional. 242 adult women.	Does not describe	Questionnaire	Does not describe	Descriptive statistics
1998^(15)^	Cross-sectional. 1,693 adult women.	Does not describe	Questionnaire	Health facility	Descriptive and inferential statistics
1998^(16)^	Cross-sectional. 155 adult women.	Convenience	Extraction Form	In a private location	Descriptive and inferential statistics
2000^(17)^	Cross-sectional. 4,643 male and female adolescents.	Randomized, Systematic	Interview	Does not describe	Descriptive and inferential statistics
2001^(18)^	Case Series. 110 documents of male and female children.	Random	Does not describe	Database	Descriptive statistics
2003^(19)^	Cohort: 4,904 adult women	Does not describe	Interview	Home	Descriptive and inferential statistics
2003^(20)^	Cross-sectional. 190 adult women.	Does not describe	Questionnaire	Does not describe	Inferential statistics
2004^(21)^	Cross-sectional. 157 children and adolescents of both sexes.	Does not describe	Questionnaire	School	Descriptive statistics
2004^(22)^	Cross-sectional. 192 children, both male and female.	Convenience	Questionnaire	School	Descriptive statistics
2004^(23)^	Cross-sectional. 500 adult women.	Random	Interview	Home	Descriptive statistics
2005^(24)^	Cross-sectional. 431 female adolescents and adults.	Does not describe	Does not describe	Does not describe	Does not describe
2006^(25)^	Cross-sectional. 512 adolescents, adults, and older individuals of both sexes.	Random	Interview	Home	Descriptive and inferential statistics
2007^(26)^	Cross-sectional. 20,274 male and female adolescents.	Does not describe	Interview	School	Inferential statistics
2007^(27)^	Cross-sectional. 1,266 adult women.	Does not describe	Questionnaire	Workplace	Descriptive and inferential statistics
2007^(28)^	Cross-sectional. 3,664 adult women.	Does not describe	Questionnaire	Health facility	Descriptive and inferential statistics
2007^(29)^	Cross-sectional. 1,078 older men and women.	Random	Questionnaire and interview	Home	Descriptive and inferential statistics
2008^(30)^	Cross-sectional. 883 adult women.	Random *clusters*	Questionnaire	Home	Descriptive and inferential statistics
2008^(31)^	Cross-sectional. 1,684 male and female adolescents.	Random	Questionnaire	School	Descriptive and inferential statistics
2009^(32)^	Cross-sectional and descriptive. 340 adult women.	Several stages	Questionnaire	Home	Descriptive and inferential statistics
2009^(33)^	Cross-sectional. 387 adult women.	Does not describe	Questionnaire	Health facility	Descriptive and inferential statistics
2009^(34)^	Cross-sectional. 4,411 female adolescents and adults.	Random	Questionnaire and interview	Home	Descriptive and inferential statistics
2010^(35)^	Cross-sectional. 141 female adolescents and adults.	Randomized, Systematic	Interview	Home	Descriptive statistics
2010^(36)^	Cross-sectional. 5,106 female adolescents.	Two-stage cluster randomization	Interview	Home	Descriptive and inferential statistics
2011^(37)^	Cross-sectional. 1,502 adult and older women	Randomized, Systematic	Questionnaire and interview	Home	Descriptive statistics
2011^(38)^	Cross-sectional. 1,051 older men and women.	Does not describe	Questionnaire	Home	Descriptive and inferential statistics
2011^(39)^	Cross-sectional. 765 adult women.	Does not describe	Questionnaire	Does not describe	Descriptive and inferential statistics
2011^(40)^	Cross-sectional. 7,432 adult women.	Does not describe	Questionnaire	Does not describe	Descriptive and inferential statistics
2011^(41)^	Cross-sectional. 1,296 adolescents and adults of both sexes.	Randomized, Systematic	Questionnaire	Home	Descriptive and inferential statistics
2012^(42)^	Population-based survey. 597 adult females.	Random	Questionnaire	Home	Descriptive and inferential statistics
2012^(43)^	Cross-sectional. 236 older men and women.	Convenience	Interview	Home	Does not describe
2012^(44)^	Cross-sectional. 2,000 older men and women.	Cluster in two stages	Questionnaire and interview	Home	Descriptive and inferential statistics
2012^(45)^	Cross-sectional. 1,296 adolescents and adults of both sexes.	Randomized, Systematic	Questionnaire	Home	Descriptive and inferential statistics
2013^(46)^	Cross-sectional. 235 couples of both sexes.	Conglomerates	Questionnaire	School	Descriptive and inferential statistics
2013^(47)^	Cross-sectional. 300 female adolescents and female adults.	Simple random	Questionnaire	Private location	Descriptive and inferential statistics
2013^(48)^	Cross-sectional. 713 occurrences of children of both male and female sexes.	Does not describe	Extraction form	Database	Descriptive statistics
2014^(49)^	Cross-sectional. 1,273 adult women.	Does not describe	Interview	Private location	Descriptive and inferential statistics
2014^(50)^	Cross-sectional. 2,869 male and female adolescents and adults.	Random	Questionnaire	Home	Descriptive and inferential statistics
2014^(51)^	Cross-sectional. 392 female adolescents and adults.	Several stages	Questionnaire	Place of preference	Descriptive and inferential statistics
2014^(52)^	Cross-sectional. 345 adult women.	Random	Questionnaire and interview	Private location	Descriptive and inferential statistics
2014^(53)^	Cross-sectional. 380 adult women.	Random	Questionnaire and interview	Home	Descriptive statistics
2014^(54)^	Cross-sectional. 897 older men and women.	Two stages	Questionnaire	Home	Descriptive and inferential statistics
2015^(55)^	Cross-sectional and descriptive. 314 adult females.	Does not describe	Questionnaire	Does not describe	Descriptive and inferential statistics
2015^(56)^	Cohort: 973 children, adolescents, and adults, both male and female.	Does not describe	Interview	Health facility	Descriptive statistics
2015^(57)^	Cross-sectional. 220 adult women.	Does not describe	Questionnaire	Does not describe	Descriptive and inferential statistics
2015^(58)^	Cross-sectional. 493 children and adolescents of both sexes.	Several stages	Questionnaire	School	Inferential statistics
2015^(59)^	Cross-sectional. 4,131 male and female children and adolescents.	Multi-stage conglomerates	Questionnaire	School	Descriptive and inferential statistics
2016^(60)^	Cross-sectional. 310 adult women.	Does not describe	Questionnaire	Home	Descriptive and inferential statistics
2016^(61)^	Cross-sectional. 694 incidents involving adult females.	Does not describe	Does not describe	Does not describe	Does not describe
2016^(62)^	Cross-sectional. 355 adult women.	Randomized, Systematic	Questionnaire	Place of preference	Descriptive and inferential statistics
2017^(63)^	Cohort: 455 adult women	Does not describe	Cell examination and questionnaire	Does not describe	Descriptive statistics
2017^(64)^	Cross-sectional. 76 adult women.	Several stages	Interview	Does not describe	Descriptive and inferential statistics
2017^(65)^	Case series. 471 Police Reports of violence against adult women	Does not describe	Extraction form	Database	Descriptive and spatial statistics
2018^(66)^	Cross-sectional. 200 older men and women.	Does not describe	Questionnaire	Private Location	Descriptive and inferential statistics
2018^(67)^	Cohort: 455 adult women	Does not describe	Questionnaire	Does not describe	Descriptive and inferential statistics
2018^(68)^	Case-control study. 214 adult women	Does not describe	Questionnaire	Health Establishment	Descriptive and inferential statistics
2018^(69)^	Cross-sectional. 2,381 female adolescents and adults.	Multi-stage randomization	Questionnaire and interview	Home	Descriptive and inferential statistics
2018^(70)^	Case series. 471 police reports of children, adolescents, adults, and older individuals of both sexes.	Convenience	Extraction form	Database	Does not describe
2018^(71)^	Cohort: 307 children, both male and female.	Does not describe	Questionnaire	School	Inferential statistics
2018^(72)^	Cross-sectional. 1,484 male and female adolescents and adults	Several stages	Questionnaire and interview	Home	Descriptive and inferential statistics
2018^(73)^	Cross-sectional. 800 female adolescents and adults.	Random	Interview	Private location	Descriptive and inferential statistics
2018^(74)^	Cross-sectional. 339 older men and women.	Random	Interview	Does not describe	Descriptive and inferential statistics
2019^(75)^	Cross-sectional. 409 adult women.	Consecutive	Questionnaire	Home	Descriptive and inferential statistics
2019^(76)^	Cross-sectional. 409 female adolescents and adults.	Does not describe	Questionnaire	Health facility	Descriptive and inferential statistics
2019^(77)^	Cross-sectional. 337 female and male adults	Does not describe	Interview	Home	Descriptive and inferential statistics
2019^(78)^	Cross-sectional. 442 male and female adolescents.	Does not describe	Interview	School	Descriptive and inferential statistics
2019^(79)^	Cross-sectional. 1,326 children of both sexes.	Random clusters	Questionnaire	School	Descriptive and inferential statistics
2019^(80)^	Cross-sectional. 1,320 children of both sexes.	Stratified randomization	Questionnaire	Does not describe	Inferential statistics
2019^(81)^	Cross-sectional. 980 adult women.	Randomized, Systematic	Questionnaire	Home	Does not describe
2019^(82)^	Cross-sectional. 301 adult women.	Simple random	Questionnaire	Does not describe	Descriptive and inferential statistics
2019^(83)^	Cross-sectional. 243 older men and women.	Does not describe	Questionnaire	Home	Descriptive and inferential statistics
2019^(84)^	Cross-sectional. 116 adult women.	Simple random	Questionnaire	Home	Does not describe
2019^(85)^	Cross-sectional. 1,000 adult males.	Multi-stage randomization	Questionnaire	Home	Descriptive and inferential statistics
2019^(86)^	Cross-sectional. 1,416 children of both sexes.	Simple random	Questionnaire	Private Location	Descriptive and inferential statistics
2019^(87)^	Cross-sectional. 137 older men and women.	Does not describe	Questionnaire	Does not describe	Does not describe
2020^(88)^	Household survey. 729 female adolescents.	Does not describe	Interview	Home	Descriptive and inferential statistics
2020^(89)^	Cross-sectional. 1,429 adult women.	Probabilistic	Interview	Home	Inferential statistics
2020^(90)^	Cross-sectional. 538 female adolescents and adults.	Cluster in several stages	Questionnaire	Health facility	Descriptive and inferential statistics
2020^(91)^	Cross-sectional. 3,409 children and adult caregivers, both male and female.	Several stages	Questionnaire	Home	Descriptive statistics
2020^(92)^	Cross-sectional. 776 adult women.	Several stages	Questionnaire	Home	Does not describe
2020^(93)^	Cross-sectional. 246 older men and women.	Random	Questionnaire and interview	Home	Descriptive and inferential statistics
2020^(94)^	Cross-sectional. 213,782 male and female adolescents.	Does not describe	Does not describe	Database	Descriptive statistics
2020^(95)^	Cross-sectional. 3,966 adult women.	Does not describe	Questionnaire	Home	Descriptive and inferential statistics
2020^(96)^	Cross-sectional. 250 female adolescents and adults.	Simple random	Interview	Home	Descriptive and inferential statistics
2020^(97)^	Cross-sectional. 5,440 adult women.	Random	Questionnaire	Home	Descriptive and inferential statistics
2020^(98)^	Cross-sectional. 5,440 adult women.	Random	Questionnaire	Home	Descriptive and inferential statistics
2020^(99)^	Cross-sectional. 152 older men and women.	Simple random	Questionnaire and form	Health facility	Descriptive and inferential statistics
2021^(100)^	Cross-sectional. 280 female and male adults	Does not describe	Questionnaire	Does not describe	Descriptive and inferential statistics
2021^(101)^	Cross-sectional. 677 adult women.	Random	Questionnaire	Private location	Descriptive and inferential statistics
2021^(102)^	Cross-sectional. 373 adult women.	Several stages	Questionnaire	Health facility	Descriptive and inferential statistics
2021^(103)^	Cross-sectional. 655 male and female adolescents and adults.	Does not describe	Questionnaire	School	Descriptive and inferential statistics
2021^(104)^	Cross-sectional. 4,943 children of both sexes.	Cluster in several stages	Questionnaire	School	Descriptive and inferential statistics
2021^(105)^	Cross-sectional. 140 adult women.	Does not describe	Questionnaire	Private location	Descriptive and inferential statistics
2021^(106)^	Cross-sectional. 290 adult women.	Randomized, Systematic	Questionnaire	Does not describe	Descriptive and inferential statistics
2021^(107)^	Cross-sectional. 1,929 male and female adolescents and adults.	Cluster	Questionnaire	Home	Descriptive and inferential statistics
2022^(108)^	Cross-sectional. 336 adult women.	Probabilistic	Questionnaire	Home	Descriptive and inferential statistics
2022^(109)^	Cross-sectional. 1,062 children and adolescents of both sexes.	Random	Questionnaire	Home	Descriptive and inferential statistics
2022^(110)^	Cross-sectional. 3,137 adult women.	Does not describe	Questionnaire	Does not describe	Descriptive and inferential statistics
2022^(111)^	Cross-sectional. 623 adult women.	Simple random	Interview	Home	Descriptive and inferential statistics
2022^(112)^	Cross-sectional. 500 female adolescents.	Random	Interview	School	Descriptive and inferential statistics
2022^(113)^	Cross-sectional. 945 adult women.	Does not describe	Questionnaire and interview	Does not describe	Inferential statistics
2022^(114)^	Cross-sectional. 44 male and female adolescents, adults, and older individuals	Does not describe	Questionnaire	Home	Descriptive and inferential statistics
2023^(115)^	Cross-sectional. 238 male and female children and adolescents.	Random	Extraction Form	Database	Descriptive and inferential statistics
2023^(116)^	Cross-sectional. 450 adults, both male and female.	Several stages	Questionnaire	Home	Descriptive and inferential statistics
2023^(117)^	Cross-sectional. 365 adult women.	Random	Questionnaire	Home	Descriptive and inferential statistics
2024^(118)^	Cross-sectional. 79,229 adult women.	Does not describe	Extraction Form	Database	Descriptive and inferential statistics
2024^(119)^	Cross-sectional. 100 male and female children.	Snowball	Questionnaire	Home	Inferential statistics
2025^(120)^	Cross-sectional. 100 male and female children.	Snowball	Questionnaire	Home	Inferential statistics
2025^(121)^	707 female adolescents, adults and older people	Random	Interview	Home	Inferential statistics
2025^(122)^	Cohort: 933 adult women	Convenience	Interview	Home	Inferential statistics

The total number of participants included in the studies was 431,991 individuals (Chart 2). The population distribution was as follows: 247,160 adolescents (57.20%); 131,347 adults (30.40%); 19,582 adolescents and adults (4.53%); 13,424 children (3.11%); 6,579 older people (1.52%); 6,281 children and adolescents (1.45%); 3,409 children and adults (0.79%); 1,502 adults and older people (0.35%); 1,263 adolescents, adults and older people (0.29%); 973 children, adolescents and adults (0.23%); and 471 children, adolescents, adults and older people (0.11%). It should be noted that the distribution was presented in this way due to the absence of stratified data in some studies, which only indicated the overall number of participants.

Regarding participants’ age range, 51 studies (46.36%) included adults; 16 (14.55%), adolescents and adults; 11 (10.00%), older people; 10 (9.09%), children; eight (7.27%), adolescents; seven (6.36%), children and adolescents; three (2.73%), adolescents, adults and older people; one (0.91%), children and adults; one (0.91%), children, adolescents and adults; one (0.91%), children, adolescents, adults and older people; and one (0.91%), adults and older people (Chart 2).

Regarding the sex of the participants, 62 (56.36%) studies were conducted exclusively with women; 47 (42.73%) with people of both sexes; and one study (0.91%) included only men. Considering the total number of participants, studies including both men and women totaled 282,053 individuals (65.29%), while 148,938 (34.49%) were women and 1,000 (0.23%) were men (Chart 2).

With respect to sample definition, 41 (37.27%) studies do not describe the technique used, 21 (19.09%) adopted random sampling, and 11 (10.00%) adopted multi-stage sampling. Of the total productions analyzed, 65 (59.09%) used questionnaires, 23 (20.91%) used interviews and 10 (9.09%) combined questionnaires and interviews. Most of the population surveys were conducted in households – 47 (42.73%); 20 (18.18%) did not specify the collection location; 14 (12.73%) were carried out in schools; and 10 (9.09%), in private locations (Chart 2).

For statistical analysis, 76 (69.09%) studies used descriptive and inferential statistics, 13 (11.82%) applied only descriptive statistics, 12 (10.91%) used exclusively inferential statistics, eight (7.27%) did not describe the statistical methods, and one (0.91%) study used descriptive and spatial statistics (Chart 2).

The sociodemographic and cultural variables considered in the studies included age, sex, religion, ethnicity, education level, number and sex of children, marital status, family composition, income, occupation, and household characteristics (such as number of rooms). The variables linked to equity policies included disability, sexual orientation, gender identity, generation, and other social markers of difference, as well as aspects directly related to violence, such as history and location of occurrence, main aggressor, type of violence, and presence of disability. Finally, the variables related to health history included clinical conditions, medication use, history of illnesses, and previous pregnancy.

## DISCUSSION

In the research presented, a profile of quantitative studies with a cross-sectional design was identified, with an increase in the number of publications being observed during the pandemic period, as well as a higher incidence of female victims. Regarding the participants, adolescents predominated, while, within the age range, adult females were the majority. The Asian continent accounted for the largest number of publications. In respect of the sample definition, most studies did not specify the sampling technique used. In data collection, questionnaires were predominantly used. Data was mostly collected in households. The statistical analysis used in the studies was mostly descriptive and inferential.

The choice of a quantitative approach reveals the emphasis of the studies on examining the relationships between variables as a fundamental strategy for answering the formulated questions and hypotheses^(69,123)^. From this perspective, when the object of investigation is violence, the application of quantitative instruments to population samples allows for the identification of statistical trends and the establishment of significant correlations between variables^(2,82,101)^.

Cross-sectional studies were the most frequently used methodological design in the investigations included in this review. This choice is justified, above all, by the fact that, in various situations, the researcher’s interest lies on estimating parameters of a target population, verifying the prevalence and most recurrent types of violence, as well as establishing a clinical-epidemiological profile^(85,97,113,114)^. In this sense, cross- sectional studies seek to define such parameters and formulate hypotheses about possible relationships between dependent and independent variables, considering point measurements over time^(115,124)^.

An increase in the number of publications on the subject was observed in the period 2017–2022. Specifically in the period from 2020 to 2022, the COVID-19 (SARS-CoV-2) pandemic caused multiple impacts on the health of the world’s population, including the exacerbation of situations of violence^(112)^. Social distancing, adopted as a strategy to contain the spread of the virus, contributed to the intensification of violence in its different forms, particularly affecting women, older people, children, and adolescents in different countries and social strata^(8,108)^. In Brazil and other parts of the world, increases in rates of violence against women have been recorded since the implementation of isolation and confinement measures^(3,100,111)^. Hotlines for reporting abuse showed an 84% increase in Argentina, 12% in Colombia, and 16% in Peru^(8)^, highlighting a direct impact on the victims’ quality of life. In this context, isolation, coupled with women’s socioeconomic vulnerability, exacerbated situations of injustice and social exclusion^(108,109)^.

In rural areas, research indicates that such measures have further increased the fragility of the social support network and access to support structures, which were already limited before the health crisis^(101,105)^. The suspension of group coexistence and leisure activities has compromised socialization areas, reducing opportunities to cope with and break the cycle of violence^(106,108)^.

In addition, the review included studies with children, adolescents, adults, and older people^(125-127)^. Regarding adolescents, who represented the largest contingent of participants in this study, research reveals that violence constitutes a public health challenge, resulting in approximately 227 deaths per day worldwide, in addition to numerous hospitalizations^(128,129)^. Furthermore, adolescents exhibit high vulnerability to physical, sexual, and psychological violence, neglect, and bullying^(1,7,79,80,91,97,103,130)^. In Egypt, a study showed that 15.2% of adolescents in rural areas were exposed to physical violence, while 17.8% suffered sexual violence and 7.3% were victims of both types^(88)^. In Brazil, data indicates that, between 2016 and 2020, there were 34,918 intentional violent deaths of children and adolescents aged 0 to 19 years old^(1,130)^, reinforcing the need for awareness and effective action to address the problem^(119,120)^.

Conversely, most of the research has been conducted with women. In the context of gender-based violence, defined as violence suffered by women because they are women, it is estimated that approximately one in three women (35%) worldwide have experienced physical and/or sexual violence perpetrated by partners or third parties during their lifetime^(131)^. Therefore, gender-based violence constitutes a global problem^(3,100,117,118,122)^.

In rural areas, violence is exacerbated by a context of adversity and exclusion that contributes to its invisibility^(116,118,122,130)^. Relationships permeated by male chauvinism, authoritarianism, and gender inequalities^(96,111)^, as well as lack of social support, increase the vulnerability of rural women to violence^(6,44,98)^, negatively impacting their health and well-being^(110,122,132–134)^.

In the Asian continent, where most of the research was conducted, high prevalences of violence against rural women stand out, such as in Bangladesh (82.7%)^(132)^ and India (49.5%)^(6)^. Factors such as ethnic, religious, and agrarian conflicts, poverty, inequalities, and patriarchal cultural norms contribute to the perpetuation of domestic and community violence^(6,94,96)^.

In Brazil, there is a limited number of publications on this topic, with only five studies included in this review. One of them indicates the highest occurrence of violence against women, mainly on Sundays, between 12 pm and 6 pm^(65)^. Another one connects violence to contexts marked by high socioeconomic vulnerability^(70)^. There is also a study addressing the situation of disabled people who are victims of violence in rural areas^(114)^, while another one investigates cases of rape against children and adolescents in rural areas of Alagoas^(115)^. Finally, a study presents a comprehensive characterization of the reports of violence against women in rural areas in Brazil, between 2011 and 2020^(118)^.

The application of conventional sampling techniques in rural areas is hampered by remote and difficult-to-access locations, which compromises the representativeness of the samples^(135)^. The small population, low levels of education, high illiteracy rates, heterogeneity of demographic and economic groups, as well as high logistics and transportation costs also limit the scope of these studies^(105,106,135)^. Furthermore, several studies did not specify the sampling method used^(136)^, corroborating the findings of this review.

A diversity of data collection instruments was observed, without standardization among the studies, with household questionnaires and structured interviews predominating. These instruments are widely used in rural areas because they allow for the acquisition of objective and measurable information with relative logistical ease^(75,101,102,106–108,137)^. Home-based data collection, predominant in this review, is common in national studies^(2)^. This type of data collection allows for individualized attention and an understanding of the participants’ life context^(138)^, although it may be made more difficult by the victims’ fear and the presence of the perpetrator^(94,101,121)^.

In research on violence, the choice of statistical analysis, especially descriptive and inferential statistics, proves relevant because it allows for the description of the prevalence of violence in the population, the identification of associated risk factors, and the evaluation of the impact of violence prevention programs^(99)^. This approach aligns with the findings of this review.

Regarding the variables analyzed, it is important to consider the social, economic, and cultural diversity of the rural environment^(94,97,98)^. Variables such as age, sex, education level, marital status, and occupation are essential for characterizing this population^(138)^. Economic and demographic factors, such as low levels of education, lower average monthly income, and limited access to health services, are also determining factors^(99,113)^. Cultural issues, such as traditions, values, and religious beliefs, directly influence decision-making and the level of engagement in collective activities. In many rural areas, strong bonds between friends and neighbors prevail, stemming from leisure and religious activities, requiring cultural sensitivity in the development of health initiatives^(133)^. Furthermore, aspects related to sexual orientation should be considered, as certain groups may face barriers to social acceptance and access to health services^(139)^. Therefore, collecting data on these variables in rural areas is fundamental to understanding the diversity and characteristics of rural populations, allowing for the planning of effective policies, programs, and interventions tailored to their needs^(138)^.

Violence in rural areas is a broad and multifaceted phenomenon that requires an understanding of the specificities of its occurrence^(3,139)^. This review contributes by highlighting the main methodological gaps and by stimulating the production of new studies that consider the specificities of rural areas. The findings underscore the central role of population surveys as instruments capable of capturing the complexity of violence in rural areas, pointing the way to improving surveillance and public policies for equity.

### Study Limitations

The findings of this review highlight specific characteristics of violence in rural contexts, with potential contributions to research, policy, and practice in health. Identifying vulnerable groups, such as women, adolescents, and people with disabilities, as well as factors that intensify violence, such as social isolation, socioeconomic vulnerability, and barriers to accessing health services, allows for the development of more contextualized public policies and prevention programs. The predominance of quantitative methods and household questionnaires demonstrates the feasibility of monitoring violence in rural areas, pointing to ways to strengthen surveillance and data collection. Methodological gaps and a scarcity of national studies were also evident, reinforcing the need for new research that considers the specificities of rural areas. Finally, characterizing the sociodemographic, economic, and cultural variables and the exposure of vulnerable populations provides input for context-sensitive health and education actions, strengthening awareness initiatives, contributing to the prevention and mitigation of violence in this scenario. Limitations include the failure to retrieve some records, mostly those published more than 10 years ago, and the fact that some studies did not describe the data collection techniques.

### Advances in the Field of Health and Nursing

For healthcare, especially nursing, mapping the methods used in population surveys on violence in rural areas highlights the particularities and challenges of these contexts. The creation and adaptation of data collection instruments specific to the rural reality ensures more accurate information, facilitates the identification of more vulnerable population subgroups, and supports the evaluation of programs for the prevention and fight against violence. Moreover, these findings enhance the scientific evidence base and guide more effective interventions. By involving rural communities in research, nurses promote empowerment and social participation, fostering collaborative solutions to the problem of violence.

## CONCLUSION

Mapping population surveys addressing violence against people living in rural areas revealed a significant gap in studies with robust and standardized methodologies. The identified studies showed great heterogeneity regarding the instruments used, sample sizes, and methodological designs. Furthermore, the complexity of rural areas, coupled with difficult access and scarce resources, represents a challenge for conducting research in this area.

## DATA AVAILABILITY

The entire dataset supporting the results of this study is available upon request to the corresponding author.

## References

[1] Leite FMC, Garcia MTP, Cavalcante GR, Venturin B, Pedroso MRO, Souza EAG (2023). Recurrent violence against women: analysis of reported
cases. Acta Paul Enferm.

[2] Kisa S, Gungor R, Kisa A (2023). Domestic violence against women in north african and middle
eastern countries: a scoping review. Trauma Violence Abuse.

[3] Organização Mundial da Saúde (2014). Relatório Mundial sobre a prevenção da violência.

[4] Madhivanan A, Dongre AR (2022). How to reduce domestic violence against married women? a mixed
methods study from rural Tamil Nadu. J Inj Violence Res.

[5] Perez-Vincent SM, Carreras E (2022). Domestic violence reporting during the COVID-19 pandemic:
evidence from Latin America. Rev Econ Househ.

[6] Bandeira CLJ, Arboit J, Honnef F, Silva EB, Andrade A, Costa MC (2023). Violence in rural areas against disabled people from the
perspective of their families. Rev Bras Enferm.

[7] Chen X, Wu Y, Qu J (2022). Parental migration and risk of sexual assault against children in
rural China. Crime Delinq.

[8] Malta DC, Pereira CA (2023). Doenças e agravos não transmissíveis e inquéritos em
saúde. Rev Bras Epidemiol.

[9] Instituto Brasileiro de Geografia e Estatistica (2021). Rio de Janeiro.

[10] Victora CG (2022). Why do we need population health surveys?. Cad Saude Publica.

[11] Peters MDJ, Godfrey C, McInerney P, Munn Z, Tricco AC, Khalil H, Aromataris E, Lockwood C, Porritt K, Pilla B, Jordan Z (2024). JBI manual for evidence synthesis.

[12] Tricco AC, Lillie E, Zarin W, O’Brien KK, Colquhoun H, Levac D (2018). PRISMA extension for scoping reviews (PRISMA-ScR): checklist and
explanation. Ann Intern Med.

[13] Pollock D, Peters MDJ, Khalil H, McInerney P, Alexander L, Tricco AC (2023). Recommendations for the extraction, analysis, and presentation of
results in scoping reviews. JBI Evid Synth.

[14] Krishnan SP, Hilbert JC, VanLeeuwen D, Kolia R (1997). Documenting domestic violence among ethnically diverse
populations: results from a preliminary study. Fam Community Health.

[15] Kershner M, Long D, Anderson JE (1998). Abuse against women in rural Minnesota. Public Health Nurs.

[16] van Hightower NR, Gorton J (1998). Domestic violence among patients at two rural health care
clinics: prevalence and social correlates. Public Health Nurs.

[17] Hadi A (2000). Child abuse among working children in rural Bangladesh:
prevalence and determinants. Public Health.

[18] Nhundu TJ, Shumba A (2001). The nature and frequency of reported cases of teacher perpetrated
child sexual abuse in rural primary schools in Zimbabwe. Child Abuse Negl.

[19] Sabarwal S, Santhya KG, Jejeebhoy SJ (2014). Women’s autonomy and experience of physical violence within
marriage in rural India: evidence from a prospective study. J Interpers Violence.

[20] Bhuiya A, Sharmin T, Hanifi SM (2003). Nature of domestic violence against women in a rural area of
Bangladesh: implication for preventive interventions. J Health Popul Nutr.

[21] Hilarski C, Dulmus CN, Theriot MT, Sowers KM (2004). Bully-victimization related to gender and grade
level. J Evid Based Soc Work.

[22] Dulmus CN, Theriot MT, Sowers KM, Blackburn JA (2004). Student reports of peer bullying victimization in a rural
school. Stress Trauma Crisis Int J.

[23] Jain D, Sanon S, Sadowski L, Hunter W (2004). Violence against women in India: evidence from rural Maharashtra,
India. Rural Remote Health.

[24] Malcoe LH, Carson EA, Myers OB (2005). Intimate partner violence against rural native american women:
prevalence and socioeconomic risk factors. Am J Epidemiol.

[25] Thurston WE, Patten S, Lagendyk LE (2006). Prevalence of violence against women reported in a rural health
region. Can J Rural Med.

[26] Marquart BS, Nannini DK, Edwards RW, Stanley LR, Wayman JC (2007). Prevalence of dating violence and victimization: regional and
gender differences. Adolescence.

[27] Denham AC, Frasier PY, Hooten EG, Belton L, Newton W, Gonzalez P (2007). Intimate partner violence among Latinas in eastern North
Carolina. Violence Against Women.

[28] Coker AL, Flerx VC, Smith PH, Whitaker DJ, Fadden MK, Williams M (2007). Partner violence screening in rural health care
clinics. Am J Public Health.

[29] Gómez Ricárdez LA, Rodríguez Abrego G, Krug Llamas E (2007). Prevalencia y factores asociados a violencia familiar en adultos
mayores de Ocozocoautla (Chiapas, México). Rev Esp Geriatr Gerontol.

[30] Vung ND, Ostergren P-O, Krantz G (2008). Intimate partner violence against women in rural
Vietnam-different socio-demographic factors are associated with different
forms of violence. BMC Public Health.

[31] Yen CF, Yang MS, Yang MJ, Su YC, Wang MH, Lan CM (2008). Childhood physical and sexual abuse:prevalence and correlates
among adolescents living in rural. Child Abuse Negl.

[32] Hoque ME, Hoque M, Kader SB (2009). Prevalence and experience of domestic violence among rural
pregnant women in KwaZulu-Natal, South Africa. South Afr J Epidemiol Infect.

[33] Ntaganira J, Muula A, Siziya S, Stoskopf C, Rudatsikira E (2009). Factors associated with intimate partner violence among pregnant
rural women in Rwanda. Rural Remote Health.

[34] Dalal K, Rahman F, Jansson B (2009). Wife abuse in rural Bangladesh. J Biosoc Sci.

[35] Sarkar M (2010). A study on domestic violence against adult and adolescent females
in a rural area of west bengal. Indian J Community Med.

[36] Alam N, Roy SK, Ahmed T (2010). Sexually harassing behavior against adolescent girls in rural
Bangladesh: implications for achieving millennium development
goals. J Interpers Violence.

[37] Seedhom AE (2012). El-Minia governorate.

[38] Abdel RTT, El Gaafary MM (2012). Elder mistreatment in a rural area in Egypt. Geriatr Gerontol Int.

[39] Hayati EN, Högberg U , Hakimi M, Ellsberg MC, Emmelin M (2011). Behind the silence of harmony: risk factors for physical and
sexual violence among women in rural Indonesia. BMC Womens Health.

[40] Mogford E (2011). When status hurts: dimensions of women’s status and domestic
abuse in rural Northern India. Violence Against Women.

[41] Lamichhane P, Puri M, Tamang J, Dulal B (2011). Women’s status and violence against young married women in rural
Nepal. BMC Womens Health.

[42] Parmar P, Agrawal P, Greenough PG, Goyal R, Kayden S (2012). Sexual violence among host and refugee population in Djohong
District, Eastern Cameroon. Glob Public Health.

[43] Mackay-Barr M, Csiernik R (2012). An exploration of elder abuse in a rural Canadian
community. Critical Social Work.

[44] Wu L, Chen H, Hu Y, Xiang H, Yu X, Zhang T (2012). Prevalence and associated factors of elder mistreatment in a
rural community in People’s Republic of China: a cross-sectional
study. PLoS One.

[45] Puri M, Frost M, Tamang J, Lamichhane P, Shah I (2012). The prevalence and determinants of sexual violence against young
married women by husbands in rural Nepal. BMC Res Notes.

[46] Saile R, Neuner F, Ertl V, Catani C (2013). Prevalence and predictors of partner violence against women in
the aftermath of war: a survey among couples in northern
Uganda. Soc Sci Med.

[47] Balogun MO, Fawole OI, Owoaje ET, Adedokun B (2013). Experience and attitude of rural women to IPV in
Nigeria. Z Gesundhwiss.

[48] Fattah KN, Kabir ZN (2013). No place is safe: sexual abuse of children in rural
Bangladesh. J Child Sex Abuse.

[49] Falb KL, Annan J, Kpebo D, Gupta J (2014). Reproductive coercion and intimate partner violence among rural
women in Côte d’Ivoire: a cross-sectional study. Afr J Reprod Health.

[50] Hossain M, Zimmerman C, Kiss L, Kone D, Bakayoko-Topolska M, Manan DK (2014). Men’s and women’s experiences of violence and traumatic events in
rural Cote d’Ivoire before, during and after a period of armed
conflict. BMJ Open.

[51] Nyamhanga T, Frumence G (2014). Intimate partner violence to HIV sexual risk among married rural
women in Tarime, Tanzania. Afr J Midwifery Womens Health.

[52] Grose RG, Grabe S (2014). The explanatory role of relationship power and control in
domestic violence against women in Nicaragua: a feminist psychology
analysis. Violence Against Women.

[53] Rajini S, Vell CK, Senthil S (2014). Prevalence of domestic violence and health seeking behavior among
women in rural community of puducherry: a cross sectional
study. Int J Cur Res Rev.

[54] Chokkanathan S (2014). Factors associated with elder mistreatment in rural Tamil Nadu,
India: a cross-sectional survey. Int J Geriatr Psychiatry.

[55] Ashimi AO, Amole TG (2015). Prevalence and predictors for domestic violence among pregnant
women in a rural community Northwest, Nigeria. Niger Med J.

[56] Ashimi A, Amole T, Ugwa E (2015). Reported Sexual violence among women and children seen at the
gynecological emergency unit of a rural tertiary health facility, Northwest
Nigeria. Ann Med Health Sci Res.

[57] Cárdenas SD, Vergara KMA, Martínez FG (2015). Domestic violence and risk factors in women of African descent of
the city of Cartagena. Rev Clin Med Fam.

[58] Robalino IG (2015). Factores que influyen en la prevalencia de bullying en
estudiantes de los colegios rurales del Cantón Cuenca, Azuay,
2014. Rev Fac Cienc Med Univ Cuenca.

[59] Li Q, Zhong Y, Chen K, Zhong Z, Pan J (2015). Identifying risk factors for child neglect in rural areas of
western China. Child Care Health Dev.

[60] Chinnakali P, George J, Nair D, Premkumar NR, Saravanan N, Roy G (2016). The prevalence of domestic violence and its associated factors
among married women in a rural area of Puducherry, South
India. J Family Med Prim Care.

[61] Hosna AU, Talab A, Chowdhuary SM, Hossain J (2016). Epidemiology of non-fetal violence against women in the rural
area of Bangladesh. Inj Prev.

[62] Sapkota D, Bhattarai S, Baral D, Pokharel PK (2016). Domestic violence and its associated factors among married women
of a village development committee of rural Nepal. BMC Res Notes.

[63] Young CR, Kaida A, Kabakyenga J, Muyindike W, Martin JN, Hunt PW (2017). Prevalence and risk factors for intimate partner violence in
women living with HIV in Uganda. Open Forum Infect Dis.

[64] Ifeanyi-Obi CC, Agumagu AC, Iromuanya P (2017). Socio-economic determinants of domestic violence suffered by
rural women crop farmers in Imo State. J Agric Ext.

[65] Bueno ALM (2017). A geoepidemiologia e o lugar: espaços de sentido para as violências
contra mulheres rurais do rio grande do sul [tese].

[66] El-Khawaga G, Eladawi N, Abdel-Wahab F (2021). Abuse of rural elders in Mansoura Districts, Dakahlia, Egypt:
prevalence, types, risk factors, and lifestyle. J Interpers Violence.

[67] Young CR, Kaida A, Kabakyenga J, Muyindike W, Musinguzi N, Martin JN (2018). Prevalence and correlates of physical and sexual intimate partner
violence among women living with HIV in Uganda. PLoS One.

[68] Krause H, Ng SK, Singasi I, Kabugho E, Natukunda H, Goh J (2019). Incidence of intimate partner violence among Ugandan women with
pelvic floor dysfunction. Int J Gynaecol Obstet.

[69] Munro-Kramer ML, Scott N, Boyd CJ, Veliz PT, Murray SM, Musonda G (2018). Postpartum physical intimate partner violence among women in
rural Zambia. Int J Gynaecol Obstet.

[70] Bueno ALM, Lopes MJM (2018). Rural women and violence: readings of a reality that approaches
fiction. Ambiente Soc.

[71] Russo JN, Griese ER, Bares VJ (2018). Examining the prevalence and impact of peer victimization and
social support for rural youth. S D Med.

[72] Hou F, Cerulli C, Wittink MN, Caine ED, Qiu P (2018). Using confirmatory factor analysis to explore associated factors
of intimate partner violence in a sample of Chinese rural women: a
cross-sectional study. BMJ Open.

[73] Naved RT, Mamun MA, Parvin K, Willan S, Gibbs A, Yu M (2018). Magnitude and correlates of intimate partner violence against
female garment workers from selected factories in Bangladesh. PLoS One.

[74] Yadav UN, Tamang MK, Paudel G, Kafle B, Mehta S, Sekaran V (2018). The time has come to eliminate the gaps in the under-recognized
burden of elder mistreatment: a community-based, cross-sectional study from
rural eastern Nepal. PLoS One.

[75] Black E, Worth H, Clarke S, Obol JH, Akera P, Awor A (2019). Prevalence and correlates of intimate partner violence against
women in conflict affected northern Uganda: a cross-sectional
study. Confl Health.

[76] Clarke S, Richmond R, Black E, Fry H, Obol JH, Worth H (2019). Intimate partner violence in pregnancy: a cross-sectional study
from post-conflict northern Uganda. BMJ Open.

[77] Ogbonnaya IN, Wanyenze RK, Reed E, Silverman JG, Kiene SM (2020). Prevalence of and risk factors for intimate partner violence in
the first 6 months following HIV Diagnosis among a population-based sample
in rural Uganda. AIDS Behav.

[78] Katz AJ, Hensel DJ, Hunt AL, Zaban LS, Hensley MM, Ott MA (2019). Only yes means yes: sexual coercion in rural adolescent
relationships. J Adolesc Health.

[79] Wu PP, He M, Yang J, Wang D, Shi L, Lin SL (2019). Current status of neglect among children aged 3-6 years in rural
areas of Urumqi, China and risk factors for child neglect. ZDD Er Ke Za Zhi.

[80] Jiang H, Hu H, Zhu X, Jiang H (2019). Effects of school-based and community-based protection services
on victimization incidence among left-behind children in
China. Child Youth Serv Rev.

[81] Sambiavi S, Deswal BS, Ray S, Singh M (2019). Prevalence and associated factors of domestic violence against
married rural women of Gurugram, Haryana. Int J Community Med Public Health.

[82] Gupta RK, Kumari R, Singh P, Langer B (2019). Domestic violence: a community based cross sectional study among
rural married females in North West India. JK Sci.

[83] Ramalingam A, Sarkar S, Premarajan KC, Rajkumar RP, Subrahmanyam DK (2019). Prevalence and correlates of elder abuse: a cross-sectional,
community-based study from rural Puducherry. Natl Med J India.

[84] Cherian AG, Ram A, Victor CP, Christy H, Hembrom S, Mohan VR (2019). Domestic violence and its determinants among 15-49-year-old women
in a rural block in South India. Indian J Community Med.

[85] Malik JS, Nadda A (2019). A cross-sectional study of gender-based violence against men in
the rural area of Haryana, India. Indian J Community Med.

[86] Haque MA, Janson S, Moniruzzaman S, Rahman AKMF, Islam SS, Mashreky SR (2019). Children’s exposure to physical abuse from a child perspective: a
population-based study in rural Bangladesh. PLoS One.

[87] Kołodziejczak S, Terelak A, Bulsa M (2019). Domestic violence against seniors in rural areas of West
Pomerania, Poland. Ann Agric Environ Med.

[88] El-Gazzar AF, Aziz MM, Mohammed HM, Elgibaly O, Darwish MM (2020). Spousal violence and its determinants among married adolescent
girls in Upper Egypt. J Egypt Public Health Assoc.

[89] Jansen NA, Agadjanian V (2020). Polygyny and intimate partner violence in
Mozambique. J Fam Issues.

[90] Chilanga E, Collin-Vezina D, Khan MN, Riley L (2020). Prevalence and determinants of intimate partner violence against
mothers of children under-five years in Central Malawi. BMC Public Health.

[91] Yang C, Liu X, Yang Y, Huang X, Song Q, Wang Y (2020). Violent disciplinary behaviours towards left-behind children in
20 counties of rural China. Child Youth Serv Rev.

[92] Pundkar RD (2020). A prospective study to find the prevalence of domestic violence
against married females of rural India. Int J Community Med Public Health.

[93] Sembiah S, Dasgupta A, Taklikar CS, Paul B, Bandyopadhyay L, Burman J (2020). Elder abuse and its predictors: a cross-sectional study in a
rural area of West Bengal, eastern part of India. Psychogeriatrics.

[94] Osaghae I, Bhuiyan MA, Alonge O (2020). Predictors of non-fatal violence or assault among adolescents in
rural Bangladesh: cross-sectional study. BMJ Paediatr Open.

[95] Stake S, Ahmed S, Tol W, Ahmed S, Begum N, Khanam R (2020). Prevalence, associated factors, and disclosure of intimate
partner violence among mothers in rural desh. J Health Popul Nutr.

[96] Islam MS (2022). Intimate partner sexual violence against women in Sylhet,
Bangladesh: some risk factors. J Biosoc Sci.

[97] Haque MA, Moniruzzaman S, Janson S, Rahman AF, Mashreky SR, Eriksson UB (2021). Children’s exposure to psychological abuse and neglect: a
population-based study in rural Bangladesh. Acta Paediatr.

[98] Haque MA, Choudhury N, Ahmed SMT, Farzana FD, Ali M, Rahman SS (2022). Factors associated with domestic violence in rural
Bangladesh. J Interpers Violence.

[99] Pak M (2020). The prevalence and associated risk factors of elder abuse among
older people applied to the family health center in the rural district of
Turkey. Soc Work Health Care.

[100] Okedo-Alex IN, Akamike IC, Uneke CJ, Abateneh DD (2021). Community attitudes towards violence against women, and lived
experiences of family violence and abuse during childhood in rural eastern
nigeria: implications for policy and programming. Risk Manag Healthc Policy.

[101] Awolaran O, Olaolurun F, Asuzu M (2021). Experience of intimate partner violence among rural women in
Southwest, Nigeria. Afr J Reprod Health.

[102] Jatta JW, Baru A, Fawole OI, Ojengbede OA (2021). Intimate partner violence among pregnant women attending
antenatal care services in the rural Gambia. PLoS One.

[103] Keesler JM, Tucker M, Terrell B, Shipman K, Osborne R (2021). Two schools, one rural county: exploring adverse childhood
experiences among school-aged youth. J Aggress Maltreat Trauma.

[104] Wan G, Li L, Gu YA (2021). National study on child abuse and neglect in rural China: does
gender matter?. J Fam Violence.

[105] Divakaran SD, Girija N, Purandaran VB, Samadarsi R, Bindhu A, Jose R (2021). Prevalence of domestic violence among married en doing unskilled
manual work in a rural area of Trivandrum district. Int J Community Med Public Health.

[106] Mundodan JM, Lamiya KK, Haveri SP (2021). Prevalence of spousal violence among married women in a rural
area in North Kerala. J Family Med Prim Care.

[107] Karim R, Rahman H, Rahman S, Habib TZ, Swahnberg K (2021). Gender differences in marital violence: a cross-ethnic study
among Bengali, Garo, and Santal communities in rural
Bangladesh. PLoS One.

[108] Romahani S, Rahman MM (2022). Prevalence and factors associated with intimate partner violence
during Covid-19 pandemic in rural Samarahan, Sarawak. J Public Hlth Dev.

[109] Zhang H, Li Y, Shi R, Dong P, Wang W (2021). Prevalence of child maltreatment during the COVID-19 pandemic: a
cross-sectional survey of rural Hubei, China. Br J Soc Work.

[110] Li X, Wang J (2022). Continuity and change: violations of private patriarchal
practices and domestic violence against rural wives in China. Health Care Women Int.

[111] Kanagarajan P, Bharati DR, Sharadha MP, Lokeshmaran A, Boratne AV (2021). A community-based survey on evaluation of prevalence of domestic
violence against women in the rural area of Pondicherry. CHRISMED J Health Res.

[112] Vinay Kumar N, Malik JS, Sachdeva A, Kumar M, Kumar H (2022). Socio demographic determinants of violence among school-going
adolescent girls in a rural area of North India: a cross-sectional
study. J Family Med Prim Care.

[113] Richardson RA, Haight SC, Hagaman A, Sikander S, Maselko J, Bates LM (2022). Social support and intimate partner violence in rural Pakistan: a
longitudinal investigation of the bi-directional
relationship. SSM Popul Health.

[114] Costa MC, Silva EB, Jantsch LB, Colomé ICS, Defendi T (2022). Pessoas com deficiência em situações de violência no contexto da
ruralidade. Rev Baiana Enferm.

[115] Nascimento FL (2023). Crimes mal-ditos: estupros de crianças e adolescentes nas zonas
rurais de Alagoas (Brasil). Mundo Agrar.

[116] Idowu A, Israel OK, Obisesan OI, Ogunmodede O, Babayeju O, Abogunloko R (2023). Gender-based violence in a rural Nigerian community during the
COVID-19 era: a call for policy action. PAMJ One Health.

[117] Sambo MN, Jibril MB, Sulaiman H (2023). Perception, and experience of domestic violence among women in a
rural community in Kaduna State, Nigeria. Niger Med J.

[118] Stochero L, Pinto LW (2024). Caracterização das notificações de violência contra as mulheres
que vivem em contextos rurais no Brasil, de 2011 a 2020. Rev Bras Epidemiol.

[119] Ahad MA, Parry YK, Willis E, Ullah S (2024). Child laborers’ exposure to neglect in rural Bangladesh:
prevalence and risk factors. Child Indic Res.

[120] Ahad MA, Parry YK, Willis E, Ullah S, Ankers M (2025). Child laborers’ exposure to physical maltreatment in rural
Bangladesh: prevalence and risk factors. Asian J Criminol.

[121] Lowe H, Utumapu MF, Tevaga P, Ene P, Mannell J (2025). Disability and intimate partner violence experience among women
in rural Samoa: a cross-sectional analysis. Disabil Health J.

[122] Collins A, Maselko J, Hagaman A, Bates L, Haight SC, Kachoria AG (2025). Disability severity and risk of new or recurrent intimate partner
violence : evidence from a cohort study in rural Pakistan. Disabil Health J.

[123] Creswell JW (2007). Projeto de pesquisa: métodos qualitativo, quantitativo e misto.

[124] Rouquayrol MZ, Gurgel M (2018). Rouquayrol: epidemiologia e saúde.

[125] Brasil. Lei nº 8.069, de 13 de julho de 1990 (1990). 16 jul.

[126] Brasil. Lei Federal nº 10.741, de 1º de outubro de 2003 (2003). Dispõe sobre o Estatuto da Pessoa Idosa e dá outras
providências.

[127] Organização das Nações Unidas (2023). Organização das Nações Unidas.

[128] Organização das Nações Unidas (2006). Relatório sobre o estudo das Nações Unidas sobre a violência contra
crianças.

[129] Farias ACN, Barros MBSC, Araújo WJS, Silva ACC, Oliveira SRDE, Santos TA (2022). Factors associated with violence with adolescents in the school
context: an integrative review. RSD.

[130] Fernandes GC, Costa JVR, Oliveira CJB, Oliveira TRN, Vieira TS, Alves PMR (2020). Violence against children and adolescents residents in a rural
area in the state of Minas Gerais. RAS.

[131] Organização Pan-Americana da Saúde (2022). Violência contra as mulheres.

[132] Esie P, Osypuk TL, Schuler SR, Bates LM (2019). Intimate partner violence and depression in rural Bangladesh:
accounting for violence severity in a high prevalence
setting. SSM Popul Health.

[133] Bicalho ACS, Santos AJC, Silva GOM, Costa LS, Oliveira NG, Nascimento TS (2023). Violência doméstica em professores da rede pública estadual
durante a COVID-19. J Bras Psiquiatr.

[134] Ranzani CM, Silva SC, Hino P, Taminato M, Okuno MFP, Fernandes H (2023). Profile and characteristics of violence against older adults
during the COVID-19 pandemic. Rev Lat Am Enfermagem.

[135] Dantas MNP, Souza DLB, Souza AMG, Aiquoc KM, Souza TA, Barbosa IR (2020). Factors associated with poor access to health services in
Brazil. Rev Bras Epidemiol.

[136] Oliveira MORD, Luce FB, Sampaio CH, Perin MG, Santini FDO, Santos MJD (2017). Análise da qualidade dos artigos científicos da área de marketing
publicados no Brasil. REAd.

[137] Marconi MA, Lakatos MA (2017). Fundamentos de metodologia científica.

[138] Fausto MCR, Almeida PF, Bousquat A, Lima JG, Santos AM, Seidl H (2023). Atenção Primária à Saúde em municípios rurais remotos
brasileiros: contexto, organização e acesso à atenção integral no Sistema
Único de Saúde. Saude Soc.

[139] Batista MLP, Macêdo EM, Bezerra AKL, Silva AJ, Barros RFM (2023). Comunidade rural do Nordeste brasileiro: um cenário de reflexão
para a formulação de políticas de desenvolvimento local e empreendedorismo
sustentável. Rev Adm Pública.

