# Supplementary material for: Methodologies for population surveys on violence in rural areas: a scoping review
Source: Rev Esc Enferm USP. 2026 Feb 27;60:e20250239. doi: 10.1590/1980-220X-REEUSP-2025-0239en (PMC12991414; doi:10.1590/1980-220X-REEUSP-2025-0239en)
Supplement: Translation

# Translation: Metodologias de inquéritos populacionais referentes às violências em áreas rurais: revisão de escopo

**DOI:** 10.1590/1980-220X-REEUSP-2025-0239pt

**Authors:** Carmem Layana Bandeira Bervig, Marta Cocco, Jaqueline Arboit, Ethel Bastos da Silva, Cristiane Cardoso de Paula, Manuel Gaspar da Silva Lisboa

**Affiliations:** 1 Universidade Federal do Rio Grande do Sul, Escola de Enfermagem, Porto Alegre, RS, Brasil.; 2 Universidade Federal de Santa Maria, Departamento de Ciências da Saúde, Palmeira das Missões, RS, Brasil.; 3 Universidade Federal de Santa Maria, Centro de Ciências da Saúde, Departamento de Enfermagem, Santa Maria, RS, Brasil.; 4 Universidade Nova de Lisboa, Faculdade de Ciências Sociais e Humanas, Departamento de Sociologia, Lisboa, Portugal.

**Keywords:** Violência, Saúde da População Rural, Inquéritos e Questionários, Revisão de Escopo

## Abstract

**Objetivo:**

Examinar os métodos empregados em inquéritos populacionais referentes à epidemiologia das violências contra pessoas residentes em áreas rurais.

**Método:**

Revisão de escopo, conduzida segundo o método JBI e relatada conforme o PRISMA-ScR. O protocolo foi registrado no OSF em agosto de 2022 (DOI https://doi.org/10.17605/OSF.IO/WG6PU). As buscas ocorreram de julho a setembro de 2022, com atualização em 27 de abril de 2025, por meio do mapeamento de palavras-chave e termos controlados, guiado pelo acrônimo População, Conceito e Contexto. As fontes de dados incluíram MEDLINE, Web of Science, LILACS, Scopus, EMBASE, CINAHL, Cochrane Library, literatura cinzenta e estudos não publicados. Dois revisores independentes realizaram a seleção dos estudos.

**Resultados:**

Foram incluídos 110 estudos: 89,09% (n = 98) com delineamento transversal; 46,36% (n = 51) com adultos; e 56,36% (n = 62) com pessoas do sexo feminino. Em 37,27% (n = 41) dos estudos, não foi descrita a técnica de amostragem; 59,09% (n = 65) utilizaram questionários; e 69,09% (n = 76) realizaram análise estatística descritiva e inferencial.

**Conclusão:**

Observou-se heterogeneidade quanto aos instrumentos, amostragem e delineamentos metodológicos dos inquéritos.

## INTRODUÇÃO

A violência constitui um grave problema de saúde global, que demanda esforços intersetoriais e recursos individuais e institucionais, frequentemente limitados para seu enfrentamento efetivo^(1)^. Estatisticamente, trata-se de um fenômeno de difícil mensuração, dada a sua complexidade, invisibilidade social e subnotificação^(1,2)^. A Organização Mundial da Saúde define violência como o uso intencional da força ou do poder, real ou em ameaça, contra si próprio ou outros, que resulte ou possa resultar em lesão, morte, dano psicológico, deficiência de desenvolvimento ou privação^(3)^.

No contexto rural, a violência assume contornos ainda mais complexos devido à distância geográfica em relação às cidades, à ausência de transportes públicos, às barreiras de acesso aos serviços de saúde, à precariedade da infraestrutura e à falta de insumos^(4)^. Pessoas residentes nessas áreas enfrentam múltiplas formas de violência – física, psicológica, sexual e financeira – perpetradas por familiares, colegas, membros da comunidade e, por vezes, profissionais de saúde^(3-7)^. Embora também ocorra em áreas urbanas, a subnotificação é particularmente elevada no meio rural, pelas dificuldades de acesso a serviços protetivos e fatores socioculturais que naturalizam a violência^(1)^. Esses obstáculos aumentam a vulnerabilidade da população rural, reforçando a invisibilidade do fenômeno^(4)^.

Os inquéritos populacionais são instrumentos essenciais para a mensuração epidemiológica da violência, permitindo estimar prevalências, mapear fatores associados e subsidiar políticas públicas de prevenção e enfrentamento. Esses estudos oferecem informações sobre os tipos de violência, fatores de risco, consequências e impactos em diferentes contextos sociais, sendo fundamentais para compreender a dimensão do problema^(8,9)^. Importantes inquéritos nacionais, como a Pesquisa Nacional de Saúde (PNS)^(9)^, incluem a área rural em suas amostragens; contudo, raramente exploram em profundidade os desfechos em saúde e as particularidades da coleta de dados em contextos rurais no tocante à violência.

Apesar da relevância dos inquéritos, faltam sistematizações que examinem de forma abrangente os métodos empregados em inquéritos populacionais voltados à mensuração da violência em áreas rurais. Essa lacuna compromete a comparabilidade dos dados, limita o fortalecimento da base de evidências e dificulta o planejamento de ações intersetoriais e o monitoramento de políticas de equidade. Estudos de base populacional permanecem dispersos, metodologicamente heterogêneos e, muitas vezes, inadequados para captar as singularidades territoriais, culturais e de acesso da população que reside fora dos centros urbanos. Assim, os inquéritos populacionais são centrais para suprir essa carência, ao coletarem informações diretamente dos moradores, abordando diferentes formas de violência^(9,10)^, revelando padrões de violência frequentemente negligenciados em políticas públicas e na alocação de recursos para prevenção e mitigação do fenômeno^(8,9)^.

Tendo em vista o exposto, este estudo visa orientar a escolha de métodos mais adequados para a condução de inquéritos populacionais sobre violências em áreas rurais, com foco em técnicas de coleta e análise de dados que considerem variáveis sociodemográficas, culturais, econômicas e outros marcadores sociais. Dessa forma, esta revisão de escopo teve como objetivo examinar os métodos empregados em inquéritos populacionais referentes à epidemiologia das violências contra pessoas residentes em áreas rurais.

## MÉTODO

### Tipo de Estudo

Trata-se de uma *scoping review* conduzida de acordo com o método do JBI e relatada segundo as diretrizes do *Preferred Reporting Items for Systematic reviews and Meta-Analyses extension for Scoping Reviews* (PRISMA-ScR)^(11,12)^. O JBI é uma instituição internacional dedicada à síntese, transferência e implementação de evidências em saúde, reconhecida pelo rigor e atualização de seus delineamentos para revisões sistemáticas e de escopo. Assim, a escolha por esse referencial justifica-se pela solidez metodológica que confere à revisão^(11)^. Cabe destacar que o foco desta revisão recai sobre as estratégias metodológicas empregadas em inquéritos populacionais, sem adentrar na categorização dos tipos de violência abordados, por se tratar de um eixo analítico distinto.

Para seu desenvolvimento, seguiram-se as etapas: definição do objetivo, questão de revisão e critérios de seleção; planejamento e execução da estratégia de busca; seleção e extração dos dados; análise e apresentação dos dados; e sumarização das evidências mapeadas em consoância com o objetivo da revisão^(11)^.

Inicialmente consultaram-se *Open Science Framework* (OSF), *Medical Literature Analysis and Retrieval System Online* (MEDLINE), *Cochrane Database of Systematic Reviews*, PROSPERO (*International prospective register of systematic reviews*) e JBI *Evidence Synthesis*, não tendo sido identificadas revisões de escopo publicadas ou em andamento sobre inquéritos populacionais relativos à epidemiologia das violências no contexto rural. Diante disso, o protocolo de revisão foi elaborado e registrado publicamente no OSF em 20 agosto de 2022 (DOI https://doi.org/10.17605/OSF.IO/WG6PU).

### Identificação da Questão de Revisão

A questão de revisão foi estruturada com base no acrônimo PCC (População, Conceito e Contexto)^(11)^ sendo: P- pessoas em situação de violência; C- métodos de inquéritos de base populacional; e C- áreas rurais. A partir dessa combinação mnemônica, formulou-se a questão: Quais os métodos empregados em inquéritos populacionais referentes à epidemiologia das violências contra pessoas residentes em áreas rurais?

### Critérios de Seleção

Os critérios de inclusão e exclusão foram definidos conforme os elementos do acrônimo PCC. População (P): estudos com crianças, adolescentes, adultos e idosos em situação de violência, sem nenhuma restrição quanto à religião, orientação sexual, identidade de gênero, deficiência ou outros marcadores sociais. Adotaram-se as seguintes faixas etárias: crianças (até 12 anos de idade incompletos); adolescentes (12 anos completos até 18 anos incompletos); adultos (19 anos completos até 59 anos); e idosos (60 anos ou mais)^(5)^. Foram excluídos estudos centrados nas consequências da violência para a saúde, por abordarem desfechos clínicos e não aspectos metodológicos, o que não corresponde ao objetivo desta revisão. Conceito (C): estudos com métodos de inquéritos populacionais. Contexto (C): estudos que abordassem a área rural. Excluíram-se estudos que analisassem dados de áreas urbanas e rurais de forma conjunta.

Foram incluídos artigos de estudos experimentais e quase experimentais (ensaios clínicos controlados randomizados e não randomizados; estudos do tipo antes e depois; séries temporais); estudos observacionais analíticos (estudos de coortes prospectivos e retrospectivos, caso-controle, transversais analíticos); desenhos de pesquisa documental (como análise de prontuários); estudos observacionais descritivos (séries de casos, relatos de casos individuais e transversais descritivos); além de literatura cinzenta (dissertações, teses, monografias); disponíveis nos idiomas português, inglês ou espanhol. Excluíram-se estudos qualitativos, de métodos mistos, de criação ou validação de instrumentos, estudos de intervenção, notas prévias, estudos de revisão, protocolos de pesquisa e editoriais. Não foram aplicadas restrições quanto ao período de publicação.

### Estratégias de Busca

O JBI recomenda a utilização de uma estratégia de busca em três etapas, com o objetivo de recuperar estudos elegíveis, publicados e não publicados^(11)^. As buscas bibliográficas foram realizadas com a colaboração de uma bibliotecária especializada na recuperação da informação em saúde e certificada pelo JBI, que conduziu o mapeamento das palavras-chave e dos termos padronizados nos vocabulários controlados: Descritores em Ciências da Saúde (DECS), via Portal Regional da Biblioteca Virtual em Saúde (BVS); Medical Subject Healding (MESH) por meio do Pubmed; e Emtree (Embase subject headings), da base de dados EMBASE (Elsevier). Na primeira etapa, realizou- se uma busca inicial combinando palavras-chave e descritores nos títulos de assuntos médicos no MEDLINE via PubMed e no Portal da BVS, para mapear os termos relevantes. Em seguida, procedeu-se à análise dos títulos e resumos dos artigos recuperados, a fim de levantar palavras-chave e termos de índice comumente utilizados.

Na segunda etapa, foram incluídos termos adicionais extraídos dos títulos, resumos e descritores (MeSH/DeCS/Emtree) dos artigos, que foram aplicados nos campos título, resumo e assunto (Subject Headings, Mesh Terms, Emtree Terms, Keywords), permitindo a formulação de uma estratégia de busca final, aplicada na PubMed em setembro de 2022 (Quadro 1). Essa estratégia foi posteriormente adaptada às demais fontes de informação incluídas. As estratégias completas de todas as fontes e os arquivos de extração estão disponíveis publicamente no *SciELO Data* (https://doi.org/10.48331/SCIELODATA.MU1AB9), garantindo transparência e reprodutibilidade. Na terceira etapa, as listas de referências dos estudos incluídos foram examinadas de acordo com os critérios de elegibilidade, com o intuito de localizar outros estudos potencialmente relevantes para a revisão.

**Quadro 1:** Estratégia de busca no Pubmed aplicada em 23 de setembro de 2022 – Palmeira das Missões, RS, Brasil, 2023.

Search	Query	Results
#1	Search: “Violence”[mh] violence*[tiab] OR Crime*[tiab] OR Offense*[tiab] OR Abuse*[tiab] OR “Aggression”[mh] OR Aggression*[tiab] OR “Homicide”[mh] OR Homicide*[tiab] OR femicide*[tiab] OR mistreatment[tiab] Sort by: Most Recent	273,117
#2	Search: “Surveys and Questionnaires”[mh] OR Survey*[tiab] OR Nonrespondent*[tiab] OR Questionnaire*[tiab] OR Respondent*[tiab] OR “Health Surveys”[mh] OR “Health Survey”[tiab] OR “National Health Survey”[tiab] OR Prevalence[mh] OR Prevalence*[tiab] OR Incidence*[tiab] OR Epidemiology[mh] OR Epidemiolog*[tiab] OR “Health Surveys”[mh] OR Health Survey*[tiab] OR “population-based survey”[tiab] OR “Epidemiologic Studies”[mh] OR “Epidemiological Studies”[tiab] OR “Epidemiological Study”[tiab] OR “Epidemiologic Study”[tiab] OR “population-based household survey”[tiab] OR “population-based survey”[tiab] OR “population-based household survey”[tiab] OR “population survey”[tiab] Sort by: Most Recent	5,493,787
#3	Search: Rural Area*[tiab] OR Rural Zone*[tiab] OR “Rural Population”[mh] OR “Medium Communities”[tiab] OR Rural Communit*[tiab] OR Rural Population*[tiab] OR Rural Settlement*[tiab] OR Rural Spatial Distribution*[tiab] OR Small Communit*[tiab] OR “peasant farmers”[tiab] OR peasant[tiab] OR cottager*[tiab] OR Rural[tiab] OR countryside[tiab] OR “agricultural area”[tiab] OR “rural environment”[tiab] OR “rural land”[tiab] OR “rural dweller”[tiab] OR “rural people”[tiab] OR “rural resident”[tiab] OR “rural society”[tiab] OR “rural worker”[tiab] OR “village people”[tiab] OR “village population”[tiab] OR “village resident”[tiab] OR villager[tiab] Sort by: Most Recent	183,615
#4	Search: #1 AND #2 AND #3 Sort by: Most Recent	2,527

Fonte: Pubmed, (Org) autora.

As fontes de dados pesquisadas incluíram: Medical Literature Analysis and Retrieval System Online (MEDLINE/PubMed), Web of Science Core Collection/Clarivate Analytics, Literatura Latino-Americana e do Caribe em Ciências da Saúde (LILACS/BVS), SciVerse Scopus/Elsevier, EMBASE/Elsevier, Cumulative Index to Nursing and Allied Health (CINAHL/EBSCO), Cochrane Library/Wiley. As fontes de estudos não publicados e literatura cinzenta abrangeram: DART-E, EthoS, Open Access Theses and Dissertations (OATD), Open Grey, Networked Digital Library of Theses and Dissertations (NDLTD), ProQuest Dissertations & Theses Global (PQDT) e Banco de Teses da Coordenação de Aperfeiçoamento de Pessoal de Nível Superior (CAPES). A busca manual de referências foi realizada por meio do Google Scholar. As buscas bibliográficas foram realizadas de julho a setembro de 2022 e atualizadas em 27 de abril de 2025, reaplicando a estratégia adaptada a cada base.

### Seleção das Fontes de Evidência

Após a busca dos estudos nas fontes de dados, todos os registros identificados foram exportados para o gerenciador de referências Endnote e, após a remoção de duplicatas, importados no *software Rayyan*. Em seguida, dois revisores independentes realizaram a triagem mediante leitura de títulos/resumos em modo cego (*Blind ON/OFF)*. Nos casos em que houve divergência, um terceiro revisor foi contatado.

A seleção foi precedida por teste piloto de 25 títulos/resumos por revisor. Após discussão da equipe e atingido o consenso de 75%, deu-se continuidade à seleção^(13)^. Para garantir a fidedignidade do processo, foi desenvolvido um *checklist* com critérios de elegibilidade. Os conflitos entre as revisoras foram dirimidos pela terceira avaliadora e, quando necessário, realizou-se contato via *e-mail* com os autores dos estudos primários para esclarecimentos. Na segunda etapa, os estudos pré-selecionados foram lidos na íntegra. Todo o processo de seleção foi documentado em fluxograma, conforme as diretrizes do PRISMA-ScR^(12)^.

### Extração dos Dados

Os dados foram extraídos dos artigos incluídos por dois revisores, de forma independente e, em casos de discordância, um terceiro revisor foi consultado. Utilizou-se formulário estruturado^(11)^ no Microsoft Excel®, previamente testado para verificar sua adequação à pergunta de revisão. Os revisores foram devidamente capacitados para a condução desta etapa. As informações extraídas abrangeram: autor(es), ano de publicação, país de origem, objetivo, população e amostra, desenho do estudo, técnicas de coleta e análise de dados, bem como variáveis sociodemográficas relacionadas às políticas de equidade – de deficiência, orientação sexual, identidade de gênero e outros marcadores sociais de diferença. A variável violência em áreas rurais foi identificada conforme a definição adotada em cada estudo, considerando critérios como autodeclaração de residência rural, classificação administrativa do território ou estratificação geográfica utilizada pelos autores originais.

### Análise e Tratamento dos Dados

As evidências coletadas foram analisadas de forma descritiva e comparativa, sendo apresentadas em tabelas e gráficos. Um resumo narrativo acompanha os resultados, relacionando os achados ao objetivo e à pergunta de revisão.

### Aspectos Éticos

Por se tratar de uma revisão de literatura, sem envolvimento direto de seres humanos e que analisou exclusivamente dados secundários disponíveis publicamente, este estudo não exigiu aprovação por Comitê de Ética em Pesquisa. O protocolo, no entanto, foi apreciado e registrado conforme as recomendações internacionais de boas práticas em revisões sistemáticas e de escopo. Destaca-se que foram respeitados os direitos autorais e a integridade das pesquisas analisadas.

## RESULTADOS

A busca resultou na identificação de 23.717 estudos. Após a remoção de 11.119 duplicatas e 584 registros não elegíveis, 12.014 estudos foram submetidos à triagem por título e resumo. Desses, 11.833 foram excluídos por não responderem à questão de revisão, restando 181 para leitura na íntegra. A partir da análise das listas de referências, três estudos adicionais foram incluídos. Na leitura completa, 22 não foram recuperados e 49 foram excluídos por não atenderem aos critérios de inclusão. Assim, 110 estudos compuseram o *corpus* analítico da revisão, conforme demonstrado no fluxograma PRISMA (Figura 1).

**Figura 1:** Fluxograma PRISMA 2020 para novas revisões sistemáticas, incluindo buscas em bases de dados e registros – Palmeira das Missões/RS, Brasil, 2025.

Legenda: *BVS (n = 542), Cochrane (n = 347), EBSCO – CINAHL e SocINDEX (n = 3601), Embase (n = 5075), ETHOS (n = 10), POPLINE (n = 99), PUBMED (n = 3089), PsycINFO (n = 200), Scopus (n = 6289), WOS (n = 4388). **Retirados antes da seleção – marcados como não elegíveis. ***Excluídos por não responderem à questão de revisão. ****Razão 1: População Razão 2: Conceito Razão 3: Contexto.

A distribuição geográfica dos estudos incluídos é apresentada na Figura 2, em escala de 0 a 20. O mapa foi elaborado na plataforma *Infogram* (versão gratuita). Quanto mais intensa a cor, maior o quantitativo de estudos por país. Observa-se predominância na Índia (n = 20), Bangladesh (n = 15), Estados Unidos (n = 12) e China (n = 11).

**Figura 2:** Distribuição geográfica dos estudos incluídos na revisão – Palmeira das Missões/RS, 2025.

A Figura 3 apresenta a distribuição temporal dos estudos, publicados entre 1997 e 2025, com maior concentração no período de 2017 a 2020. Entre 2020 e 2022, anos marcados pela pandemia de COVID-19, foram identificados 28 estudos.

**Figura 3:** Quantitativo de estudos por ano de publicação – Palmeira das Missões/RS, 2025.

Todos os estudos adotaram abordagem quantitativa. Destes, 98 (89,09%) utilizaram delineamento transversal; seis (5,45%) eram estudos de coorte; três (2,73%), série de casos; um (0,91%), caso-controle; um (0,91%), inquérito de base populacional; e uma (0,91%), pesquisa domiciliar (Quadro 2).

**Quadro 2:** Quadro com identificação dos estudos – Palmeira das Missões, RS, Brasil, 2025^(7,14–122)^.

Ano Referência	Delineamento. número de participantes. faixa etária. sexo	Técnica de amostra	Técnica coleta de dados	Local coleta dados	Análise de dados
2021^(7)^	Transversal. 1.681 crianças dos sexos masculino e feminino	Conglomerados em vários estágios	Entrevista	Escola	Estatística inferencial
1997^(14)^	Transversal. 242 adultos do sexo feminino	Não descreve	Questionário	Não descreve	Estatística descritiva
1998^(15)^	Transversal. 1.693 adultos do sexo feminino	Não descreve	Questionário	Estabele-cimento de saúde	Estatística descritiva e inferencial
1998^(16)^	Transversal. 155 adultos do sexo feminino	Conveniência	Formulário de Extração	Em local privado	Estatística descritiva e inferencial
2000^(17)^	Transversal. 4.643 adolescentes dos sexos masculino e feminino	Aleatória sistemática	Entrevista	Não descreve	Estatística descritiva e inferencial
2001^(18)^	Série de Casos. 110 documentos de crianças dos sexos masculino e feminino	Aleatória	Não descreve	Banco de dados	Estatística descritiva
2003^(19)^	Coorte. 4.904 adultos do sexo feminino	Não descreve	Entrevista	Domicílio	Estatística descritiva e inferencial
2003^(20)^	Transversal. 190 adultos do sexo feminino	Não descreve	Questionário	Não descreve	Estatística inferencial
2004^(21)^	Transversal. 157 crianças e adolescentes dos sexos masculino e feminino	Não descreve	Questionário	Escola	Estatística descritiva
2004^(22)^	Transversal. 192 crianças dos sexos masculino e feminino	Conveniência	Questionário	Escola	Estatística descritiva
2004^(23)^	Transversal. 500 adultos do sexo feminino	Aleatória	Entrevista	Domicílio	Estatística descritiva
2005^(24)^	Transversal. 431 adolescentes e adultos do sexo feminino	Não descreve	Não descreve	Não descreve	Não descreve
2006^(25)^	Transversal. 512 adolescentes, adultos e idosos dos sexos masculino e feminino	Aleatória	Entrevista	Domicílio	Estatística descritiva e inferencial
2007^(26)^	Transversal. 20.274 adolescentes dos sexos masculino e feminino	Não descreve	Entrevista	Escola	Estatística inferencial
2007^(27)^	Transversal. 1.266 adultos do sexo feminino	Não descreve	Questionário	Local de trabalho	Estatística descritiva e inferencial
2007^(28)^	Transversal. 3.664 adultos do sexo feminino	Não descreve	Questionário	Estabele-cimento de saúde	Estatística descritiva e inferencial
2007^(29)^	Transversal. 1.078 idosos dos sexos masculino e feminino	Aleatória	Questionário e entrevista	Domicílio	Estatística descritiva e inferencial
2008^(30)^	Transversal. 883 adultos do sexo feminino	Aleatória de *clusters*	Questionário	Domicílio	Estatística descritiva e inferencial
2008^(31)^	Transversal. 1.684 adolescentes dos sexos masculino e feminino	Aleatória	Questionário	Escola	Estatística descritiva e inferencial
2009^(32)^	Transversal e descritivo. 340 adultos do sexo feminino	Vários estágios	Questionário	Domicílio	Estatística descritiva e inferencial
2009^(33)^	Transversal. 387 adultos do sexo feminino	Não descreve	Questionário	Estabele-cimento de saúde	Estatística descritiva e inferencial
2009^(34)^	Transversal. 4.411 adolescentes e adultos do sexo feminino	Aleatória	Questionário e entrevista	Domicílio	Estatística descritiva e inferencial
2010^(35)^	Transversal. 141 adolescentes e adultos do sexo feminino	Aleatória sistemática	Entrevista	Domicílio	Estatística descritiva
2010^(36)^	Transversal. 5.106 adolescentes do sexo feminino	Aleatória de conglomerados em dois estágios	Entrevista	Domicílio	Estatística descritiva e inferencial
2011^(37)^	Transversal. 1.502 adultos e idosos do sexo feminino	Aleatória sistemática	Questionário e entrevista	Domicílio	Estatística descritiva
2011^(38)^	Transversal. 1.051 idosos dos sexos masculino e feminino	Não descreve	Questionário	Domicílio	Estatística descritiva e inferencial
2011^(39)^	Transversal. 765 adultos do sexo feminino	Não descreve	Questionário	Não descreve	Estatística descritiva e inferencial
2011^(40)^	Transversal. 7.432 adultos do sexo feminino	Não descreve	Questionário	Não descreve	Estatística descritiva e inferencial
2011^(41)^	Transversal. 1.296 adolescentes e adultos dos sexos masculino e feminino	Aleatória sistemática	Questionário	Domicílio	Estatística descritiva e inferencial
2012^(42)^	Inquérito de base populacional. 597 adultos do sexo feminino	Aleatória	Questionário	Domicílio	Estatística descritiva e inferencial
2012^(43)^	Transversal. 236 idosos dos sexos masculino e feminino	Conveniência	Entrevista	Domicílio	Não descreve
2012^(44)^	Transversal. 2.000 idosos dos sexos masculino e feminino	*Cluster* em dois estágios	Questionário e entrevista	Domicílio	Estatística descritiva e inferencial
2012^(45)^	Transversal. 1.296 adolescentes e adultos do sexo feminino	Aleatória sistemática	Questionário	Domicílio	Estatística descritiva e inferencial
2013^(46)^	Transversal. 235 casais dos sexos masculino e feminino	Conglomerados	Questionário	Escola	Estatística descritiva e inferencial
2013^(47)^	Transversal. 300 adolescentes do sexo feminino e adultos do sexo feminino	Aleatória simples	Questionário	Local privado	Estatística descritiva e inferencial
2013^(48)^	Transversal. 713 ocorrências de crianças dos sexos masculino e feminino	Não descreve	Formulário de extração	Banco de dados	Estatística descritiva
2014^(49)^	Transversal. 1.273 adultos do sexo feminino	Não descreve	Entrevista	Local privado	Estatística descritiva e inferencial
2014^(50)^	Transversal. 2.869 adolescentes e adultos dos sexos masculino e feminino	Aleatória	Questionário	Domicílio	Estatística descritiva e inferencial
2014^(51)^	Transversal. 392 adolescentes e adultos do sexo feminino	Vários estágios	Questionário	Local de preferência	Estatística descritiva e inferencial
2014^(52)^	Transversal. 345 adultos do sexo feminino	Aleatória	Questionário e entrevista	Local privado	Estatística descritiva e inferencial
2014^(53)^	Transversal. 380 adultos do sexo feminino	Aleatória	Questionário e entrevista	Domicílio	Estatística descritiva
2014^(54)^	Transversal. 897 idosos dos sexos masculino e feminino	Dois estágios	Questionário	Domicílio	Estatística descritiva e inferencial
2015^(55)^	Transversal e Descritivo. 314 adultos do sexo feminino	Não descreve	Questionário	Não descreve	Estatística descritiva e inferencial
2015^(56)^	Coorte. 973 crianças, adolescentes e adultos dos sexos masculino e feminino	Não descreve	Entrevista	Estabele-cimento de saúde	Estatística descritiva
2015^(57)^	Transversal. 220 adultos do sexo feminino	Não descreve	Questionário	Não descreve	Estatística descritiva e inferencial
2015^(58)^	Transversal. 493 crianças e adolescentes dos sexos masculino e feminino	Vários estágios	Questionário	Escola	Estatística inferencial
2015^(59)^	Transversal. 4.131crianças e adolescentes dos sexos masculino e feminino	Conglomerados em vários estágios	Questionário	Escola	Estatística descritiva e inferencial
2016^(60)^	Transversal. 310 adultos do sexo feminino	Não descreve	Questionário	Domicílio	Estatística descritiva e inferencial
2016^(61)^	Transversal. 694 incidentes de adultos do sexo feminino	Não descreve	Não descreve	Não descreve	Não descreve
2016^(62)^	Transversal. 355 adultos do sexo feminino	Aleatória sistemática	Questionário	Local de preferência	Estatística descritiva e inferencial
2017^(63)^	Coorte. 455 adultos do sexo feminino	Não descreve	Exame de células e questionário	Não descreve	Estatística descritiva
2017^(64)^	Transversal. 76 adultos do sexo feminino	Vários estágios	Entrevista	Não descreve	Estatística descritiva e inferencial
2017^(65)^	Série de casos. 471 Boletins de Ocorrência de violência contra adultos do sexo feminino	Não descreve	Formulário de extração	Banco de Dados	Estatística descritiva e espacial
2018^(66)^	Transversal. 200 idosos dos sexos masculino e feminino	Não descreve	Questionário	Local Privado	Estatística descritiva e inferencial
2018^(67)^	Coorte. 455 adultos do sexo feminino	Não descreve	Questionário	Não descreve	Estatística descritiva e inferencial
2018^(68)^	Estudo caso-controle. 214 adultos do sexo feminino	Não descreve	Questionário	Estabele-cimento de Saúde	Estatística descritiva e inferencial
2018^(69)^	Transversal. 2.381 adolescentes e adultos do sexo feminino	Aleatória em vários estágios	Questionário e entrevista	Domicílio	Estatística descritiva e inferencial
2018^(70)^	Série de casos. 471 boletins de ocorrência de crianças, adolescentes, adultos e idosos, sexos masculino e feminino	Conveniência	Formulário de extração	Banco de dados	Não descreve
2018^(71)^	Coorte. 307 crianças dos sexos masculino e feminino	Não descreve	Questionário	Escola	Estatística inferencial
2018^(72)^	Transversal. 1.484 adolescentes e adultos dos sexos masculino e feminino	Vários estágios	Questionário e entrevista	Domicílio	Estatística descritiva e inferencial
2018^(73)^	Transversal. 800 adolescentes e adultos do sexo feminino	Aleatória	Entrevista	Local privado	Estatística descritiva e inferencial
2018^(74)^	Transversal. 339 idosos dos sexos masculino e feminino	Aleatória	Entrevista	Não descreve	Estatística descritiva e inferencial
2019^(75)^	Transversal. 409 adultos do sexo feminino	Consecutiva	Questionário	Domicílio	Estatística descritiva e inferencial
2019^(76)^	Transversal. 409 adolescentes e adultos do sexo feminino	Não descreve	Questionário	Estabele-cimento de saúde	Estatística descritiva e inferencial
2019^(77)^	Transversal. 337 adultos dos sexos feminino e masculino	Não descreve	Entrevista	Domicílio	Estatística descritiva e inferencial
2019^(78)^	Transversal. 442 adolescentes dos sexos masculino e feminino	Não descreve	Entrevista	Escola	Estatística descritiva e inferencial
2019^(79)^	Transversal. 1.326 crianças dos sexos masculino e feminino	Aglomerados aleatórios	Questionário	Escola	Estatística descritiva e inferencial
2019^(80)^	Transversal. 1.320 crianças dos sexos masculino e feminino	Aleatória estratificada	Questionário	Não descreve	Estatística inferencial
2019^(81)^	Transversal. 980 adultos do sexo feminino	Aleatória sistemática	Questionário	Domicílio	Não descreve
2019^(82)^	Transversal. 301 adultos do sexo feminino	Aleatória simples	Questionário	Não descreve	Estatística descritiva e inferencial
2019^(83)^	Transversal. 243 idosos dos sexos masculino e feminino	Não descreve	Questionário	Domicílio	Estatística descritiva e inferencial
2019^(84)^	Transversal. 116 adultos do sexo feminino	Aleatória simples	Questionário	Domicílio	Não descreve
2019^(85)^	Transversal. 1.000 adultos do sexo masculino	Aleatória em vários estágios	Questionário	Domicílio	Estatística descritiva e inferencial
2019^(86)^	Transversal. 1.416 crianças dos sexos masculino e feminino	Aleatória simples	Questionário	Local Privado	Estatística descritiva e inferencial
2019^(87)^	Transversal. 137 idosos dos sexos masculino e feminino	Não descreve	Questionário	Não descreve	Não descreve
2020^(88)^	Pesquisa domiciliar. 729 adolescentes do sexo feminino	Não descreve	Entrevista	Domicílio	Estatística descritiva e inferencial
2020^(89)^	Transversal. 1.429 adultos do sexo feminino	Probabilística	Entrevista	Domicílio	Estatística inferencial
2020^(90)^	Transversal. 538 adolescentes e adultos do sexo feminino	*Cluster* em vários estágios	Questionário	Estabele-cimento de saúde	Estatística descritiva e inferencial
2020^(91)^	Transversal. 3.409 crianças e cuidadores adultos, dos sexos masculino e feminino	Vários estágios	Questionário	Domicílio	Estatística descritiva
2020^(92)^	Transversal. 776 adultos do sexo feminino	Vários estágios	Questionário	Domicílio	Não descreve
2020^(93)^	Transversal. 246 idosos dos sexos masculino e feminino	Aleatória	Questionário e entrevista	Domicílio	Estatística descritiva e inferencial
2020^(94)^	Transversal. 213.782 adolescentes dos sexos masculino e feminino	Não descreve	Não descreve	Banco de dados	Estatística descritiva
2020^(95)^	Transversal. 3.966 adultos do sexo feminino	Não descreve	Questionário	Domicílio	Estatística descritiva e inferencial
2020^(96)^	Transversal. 250 adolescentes e adultos do sexo feminino	Aleatória simples	Entrevista	Domicílio	Estatística descritiva e inferencial
2020^(97)^	Transversal. 5.440 adultos do sexo feminino	Aleatória	Questionário	Domicílio	Estatística descritiva e inferencial
2020^(98)^	Transversal. 5.440 adultos do sexo feminino	Aleatória	Questionário	Domicílio	Estatística descritiva e inferencial
2020^(99)^	Transversal. 152 idosos dos sexos masculino e feminino	Aleatória simples	Questionário e formulário	Estabele-cimento de saúde	Estatística descritiva e inferencial
2021^(100)^	Transversal. 280 adultos dos sexos feminino e masculino	Não descreve	Questionário	Não descreve	Estatística descritiva e inferencial
2021^(101)^	Transversal. 677 adultos do sexo feminino	Aleatória	Questionário	Local privado	Estatística descritiva e inferencial
2021^(102)^	Transversal. 373 adultos do sexo feminino	Vários estágios	Questionário	Estabele-cimento de saúde	Estatística descritiva e inferencial
2021^(103)^	Transversal. 655 adolescentes e adultos dos sexos masculino e feminino	Não descreve	Questionário	Escola	Estatística descritiva e inferencial
2021^(104)^	Transversal. 4.943 crianças dos sexos masculino e feminino	*Cluster* em vários estágios	Questionário	Escola	Estatística descritiva e inferencial
2021^(105)^	Transversal. 140 adultos do sexo feminino	Não descreve	Questionário	Local privado	Estatística descritiva e inferencial
2021^(106)^	Transversal. 290 adultos do sexo feminino	Aleatória sistemática	Questionário	Não descreve	Estatística descritiva e inferencial
2021^(107)^	Transversal. 1.929 adolescentes e adultos dos sexos masculino e feminino	*Cluster*	Questionário	Domicílio	Estatística descritiva e inferencial
2022^(108)^	Transversal. 336 adultos do sexo feminino	Probabilística	Questionário	Domicílio	Estatística descritiva e inferencial
2022^(109)^	Transversal. 1.062 crianças e adolescentes dos sexos masculino e feminino	Aleatória	Questionário	Domicílio	Estatística descritiva e inferencial
2022^(110)^	Transversal. 3.137 adultos do sexo feminino	Não descreve	Questionário	Não descreve	Estatística descritiva e inferencial
2022^(111)^	Transversal. 623 adultos do sexo feminino	Aleatória simples	Entrevista	Domicílio	Estatística descritiva e inferencial
2022^(112)^	Transversal. 500 adolescentes do sexo feminino	Aleatória	Entrevista	Escola	Estatística descritiva e inferencial
2022^(113)^	Transversal. 945 adultos do sexo feminino	Não descreve	Questionário e entrevista	Não descreve	Estatística inferencial
2022^(114)^	Transversal. 44 adolescentes, adultos e idoso dos sexos masculino e feminino	Não descreve	Questionário	Domicílios	Estatística descritiva e inferencial
2023^(115)^	Transversal. 238 crianças e adolescentes dos sexos feminino e masculino	Aleatória	Formulário de Extração	Banco de dados	Estatística descritiva e inferencial
2023^(116)^	Transversal. 450 adultos dos sexos masculino e feminino	Vários Estágios	Questionário	Domicílio	Estatística descritiva e inferencial
2023^(117)^	Transversal. 365 adultos do sexo feminino	Aleatória	Questionário	Domicílio	Estatística descritiva e inferencial
2024^(118)^	Transversal. 79.229 adultos do sexo feminino	Não descreve	Formulário de Extração	Banco de Dados	Estatística descritiva e inferencial
2024^(119)^	Transversal. 100 crianças dos sexos feminino e masculino	Bola de Neve	Questionário	Domicílio	Estatística inferencial
2025^(120)^	Transversal. 100 crianças dos sexos feminino e masculino	Bola de Neve	Questionário	Domicílio	Estatística inferencial
2025^(121)^	707 adolescentes, adultos e idosos do sexo feminino	Aleatória	Entrevista	Domicílio	Estatística inferencial
2025^(122)^	Coorte. 933 adultos do sexo feminino	Conveniência	Entrevista	Domicílio	Estatística inferencial

O total de participantes incluídos nos estudos foi de 431.991 indivíduos (Quadro 2). A distribuição populacional foi a seguinte: 247.160 adolescentes (57,20%); 131.347 adultos (30,40%); 19.582 adolescentes e adultos (4,53%); 13.424 crianças (3,11%); 6.579 idosos (1,52%); 6.281 crianças e adolescentes (1,45%); 3.409 crianças e adultos (0,79%); 1.502 adultos e idosos (0,35%); 1.263 adolescentes, adultos e idosos (0,29%); 973 crianças, adolescentes e adultos (0,23%); e 471 crianças, adolescentes, adultos e idosos (0,11%). Ressalta-se que a distribuição foi apresentada dessa forma devido à ausência de dados estratificados em alguns estudos, que apenas indicaram o quantitativo geral de participantes.

Quanto à faixa etária dos participantes, 51 estudos (46,36%) incluíram adultos; 16 (14,55%), adolescentes e adultos; 11 (10,00%), idosos; 10 (9,09%), crianças; oito (7,27%), adolescentes; sete (6,36%), crianças e adolescentes; três (2,73%), adolescentes, adultos e idosos; um (0,91%), crianças e adultos; um (0,91%), crianças, adolescentes e adultos; um (0,91%), crianças, adolescentes, adultos e idosos; e um (0,91%), adultos e idosos (Quadro 2).

Em relação ao sexo dos participantes, 62 (56,36%) estudos foram realizados exclusivamente com pessoas do sexo feminino; 47 (42,73%), com pessoas de ambos os sexos; e um estudo (0,91%) contou apenas com participantes do sexo masculino. Considerando o total de participantes, os estudos que incluíram homens e mulheres somaram 282.053 indivíduos (65,29%), enquanto 148.938 (34,49%) eram do sexo feminino e 1.000 (0,23%) do sexo masculino (Quadro 2).

Quanto à definição da amostra, 41 (37,27%) estudos não descrevem a técnica utilizada, 21 (19,09%) adotaram amostragem aleatória e 11 (10,00%), amostragem em múltiplos estágios. Do total de produções analisadas, 65 (59,09%) empregaram questionários, 23 (20,91%) utilizaram entrevistas e 10 (9,09%) combinaram questionário e entrevista. A maioria dos inquéritos populacionais foi realizada em domicílios – 47 (42,73%); 20 (18,18%) não especificaram o local de coleta; 14 (12,73%) foram desenvolvidos em escolas; e 10 (9,09%), em locais privados (Quadro 2).

Para a análise estatística, 76 (69,09%) estudos utilizaram estatística descritiva e inferencial, 13 (11,82%) aplicaram apenas a estatística descritiva, 12 (10,91%) empregaram exclusivamente a estatística inferencial, oito (7,27%) não descreveram os métodos estatísticos e um (0,91%) estudo utilizou estatística descritiva e espacial (Quadro 2).

As variáveis sociodemográficas e culturais consideradas nos estudos incluíram idade, sexo, religião, etnia, escolaridade, número e sexo de filhos, estado civil, composição familiar, renda, ocupação e características do domicílio (como número de cômodos). As variáveis vinculadas às políticas de equidade contemplaram deficiência, orientação sexual, identidade de gênero, geração e outros marcadores sociais de diferença, além de aspectos diretamente relacionados à violência, como histórico e local de ocorrência, principal agressor, tipo de violência e presença de deficiência. Por fim, as variáveis referentes à história de saúde abarcaram condições clínicas, uso de medicamentos, histórico de doenças e gravidez prévia.

## DISCUSSÃO

Na pesquisa em tela, identificou-se um perfil de estudos quantitativos com delineamento transversal, observando-se incremento no número de publicações durante o período da pandemia, bem como uma incidência maior de vítimas do sexo feminino. Quanto aos participantes, predominaram adolescentes, enquanto, na faixa etária, adultos do sexo feminino foram a maioria. O continente asiático concentrou o maior número de publicações. Quanto à definição da amostra, a maioria dos estudos não especificou a técnica de amostragem utilizada. Na coleta de dados, prevaleceu o uso de questionários. Os dados foram majoritariamente coletados em domicílios. A análise estatística adotada nos estudos foi, em sua maioria, descritiva e inferencial.

A opção pela abordagem quantitativa revela a ênfase dos estudos em examinar as relações entre variáveis como estratégia fundamental para responder às questões e hipóteses formuladas^(69,123)^. Nessa perspectiva, quando o objeto de investigação é a violência, a aplicação de instrumentos quantitativos em amostras populacionais permite identificar tendências estatísticas e estabelecer correlações significativas entre variáveis^(2,82,101)^.

Os estudos transversais configuraram o delineamento metodológico mais utilizado pelas investigações incluídas nesta revisão. Essa escolha justifica-se, sobretudo, pelo fato de que, em diversas situações, o interesse do pesquisador reside em estimar parâmetros de uma população-alvo, verificar a prevalência e os tipos de violência mais recorrentes, assim como estabelecer um perfil clínico-epidemiológico^(85,97,113,114)^. Nesse sentido, os estudos transversais buscam delimitar tais parâmetros e formular hipóteses acerca de possíveis relações entre variáveis dependentes e independentes, considerando medidas pontuais no tempo^(115,124)^.

Observou-se um aumento no número de publicações sobre a temática no período 2017–2022. Especificamente no período de 2020 a 2022, a pandemia de COVID-19 (SARS-CoV-2) provocou múltiplos impactos na saúde da população mundial, incluindo o agravamento de situações de violência^(112)^. O distanciamento social, adotado como estratégia para conter a disseminação do vírus, contribuiu para a intensificação da violência em suas diferentes expressões, afetando, de modo particular, mulheres, idosos, crianças e adolescentes, em diferentes países e estratos sociais^(8,108)^. No Brasil e em outras partes do mundo, foram registrados aumentos nos índices de violência contra as mulheres desde a implementação de medidas de isolamento e confinamento^(3,100,111)^. Linhas diretas de denúncia indicaram crescimento de 84% na Argentina, 12% na Colômbia e 16% no Peru^(8)^, evidenciando um impacto direto na qualidade de vida das vítimas. Nesse contexto, o isolamento, associado às condicões de vulnerabilidade socioeconômica das mulheres, potencializou situações de injustiças e exclusão sociais^(108,109)^.

No contexto rural, investigações apontam que tais medidas ampliaram ainda mais a fragilidade da rede de apoio social e de acesso às estruturas de suporte, já limitados antes da crise sanitária^(101,105)^. A suspensão de atividades coletivas de convivência e lazer comprometeu os espaços de socialização, reduzindo as possibilidades de enfrentamento e de ruptura do ciclo de violência^(106,108)^.

Além disso, a revisão abrangeu estudos com crianças, adolescentes, adultos e idosos^(125-127)^. Quanto aos adolescentes, que representaram o maior contingente de participantes deste estudo, pesquisas revelam que a violência constitui um desafio de saúde pública, resultando em cerca de 227 mortes por dia no mundo, além de diversas hospitalizações^(128,129)^. Ademais, os adolescentes apresentam elevada vulnerabilidade à violência física, sexual, psicológica, negligência e *bullying* ^(1,7,79,80,91,97,103,130)^. No Egito, um estudo evidenciou que 15,2% dos adolescentes em áreas rurais foram expostos à violência física, enquanto 17,8% sofreram violência sexual e 7,3% foram vítimas de ambos os tipos^(88)^. No Brasil, dados indicam que, entre 2016 e 2020, ocorreram 34.918 mortes violentas intencionais de crianças e adolescentes de 0 a 19 anos de idade^(1,130)^, reforçando a necessidade de sensibilização e ações efetivas frente à problemática^(119,120)^.

Por outro lado, a maioria das pesquisas foi conduzida com mulheres. No contexto da violência de gênero, definida como aquela sofrida por mulheres pelo fato de serem mulheres, estima- se que aproximadamente uma em cada três mulheres (35%) no mundo tenha experienciado violência física e/ou sexual perpetrada por parceiros ou terceiros ao longo da vida^(131)^. Logo, a violência de gênero configura-se como um problema global^(3,100,117,118,122)^.

No âmbito rural, a violência é potencializada pelo cenário de adversidades e exclusão que contribuem para sua invisibilidade^(116,118,122,130)^. Relações permeadas por machismo, autoritarismo e desigualdades de gênero^(96,111)^, além da ausência de apoio social, aumentam a vulnerabilidade das mulheres rurais à violência^(6,44,98)^, impactando negativamente em sua saúde e bem-estar^(110,122,132–134)^.

No continente asiático, onde a maioria das pesquisas foi realizada, destacam-se altas prevalências de violência contra mulheres rurais, como em Bangladesh (82,7%)^(132)^ e Índia (49,5%)^(6)^. Fatores como conflitos étnicos, religiosos e agrários, pobreza, desigualdades e normas culturais patriarcais contribuem para a perpetuação da violência doméstica e comunitária^(6,94,96)^.

No Brasil, observa-se um número reduzido de publicações na temática, com apenas cinco estudos incluídos nesta revisão. Um deles sinaliza a maior ocorrência de violência contra a mulher principalmente aos domingos, entre 12 h e 18 h^(65)^. Outro relaciona a violência a contextos marcados por elevada vulnerabilidade socioeconômica^(70)^. Há também um estudo que aborda a situação de pessoas com deficiência vítimas de violência no cenário rural^(114)^, enquanto outro investiga casos de estupro contra crianças e adolescentes em zonas rurais de Alagoas^(115)^. Por fim, um estudo apresenta uma caracterização abrangente das notificações de violência contra mulheres em áreas rurais do Brasil, no período de 2011 e 2020^(118)^.

A aplicação de técnicas de amostragem convencionais em áreas rurais mostra-se dificultada pela localização remota e de difícil acesso, o que compromete a representatividade das amostras^(135)^. O baixo contingente populacional, os baixos níveis de escolaridade, as altas taxas de analfabetismo, a heterogeineidade dos grupos demográficos e econômicos, bem como os altos custos logísticos e de transporte também limitam o alcance dessas pesquisas^(105,106,135)^. Ademais, diversos estudos não especificaram o método de amostragem adotado^(136)^, corroborando os achados desta revisão.

Observou-se diversidade de instrumentos de coleta, sem padronização entre os estudos, predominando questionários domiciliares e entrevistas estruturadas. Esses instrumentos são amplamente empregados em áreas rurais por permitirem a obtenção de informações objetivas e mensuráveis, com relativa praticidade logística^(75,101,102,106–108,137)^. A coleta domiciliar, predominante nesta revisão, é comum em estudos nacionais^(2)^. Esse tipo de coleta permite o atendimento individualizado e a compreensão do contexto de vida dos participantes^(138)^, embora possa ser dificultada pelo medo das vítimas e pela presença do agressor^(94,101,121)^.

Nas pesquisas sobre violência, a escolha da análise estatística, especialmente a estatística descritiva e inferencial, mostra-se relevante por possibilitar a descrição da prevalência da violência na população, a identificação de fatores de risco associados e a avaliação do impacto de programas de prevenção da violência^(99)^. Essa abordagem vai ao encontro dos achados da presente revisão.

Quanto às variáveis analisadas, destaca-se a relevância de considerar a diversidade social, econômica e cultural do meio rural^(94,97,98)^. Variáveis como idade, sexo, escolaridade, estado civil e ocupação são essenciais para caracterizar essa população^(138)^. Aspectos econômicos e demográficos, como baixa escolaridade, menor renda média mensal e acesso limitado aos serviços de saúde, também são determinantes^(99,113)^. As questões culturais, como tradições, valores e crenças religiosas, influenciam diretamente a tomada de decisões e o nível de engajamento em atividades coletivas. Em diversas áreas rurais, prevalecem laços entre amigos, vizinhos, a partir de atividades de lazer e religiosas, exigindo sensibilidade cultural na proposição de ações de saúde^(133)^. Além disso, aspectos relacionados à orientação sexual devem ser considerados, pois determinados grupos podem enfrentar barreiras quanto à aceitação social e acesso a serviços de saúde^(139)^. Diante disso, coletar dados sobre essas variáveis em áreas rurais é fundamental para compreender as diversidades e características das populações rurais, permitindo o planejamento de políticas, programas e intervenções efetivas para as suas necessidades^(138)^.

A violência em áreas rurais é um fenômeno amplo e multifacetado, que exige compreensão das especificidades de sua ocorrência^(3,139)^. Esta revisão contribui, ao evidenciar as principais lacunas metodológicas e ao estimular a produção de novos estudos que considerem as especificidades do rural. Os achados reforçam o papel central dos inquéritos populacionais como instrumentos capazes de captar a complexidade da violência em áreas rurais, apontando caminhos para o aprimoramento da vigilância e das políticas públicas de equidade.

### Limitações do Estudo

Os achados desta revisão evidenciam características específicas da violência em contextos rurais, com potenciais contribuições para a pesquisa, a política e a prática em saúde. A identificação de grupos vulneráveis, como mulheres, adolescentes e pessoas com deficiência, bem como de fatores que intensificam a violência, como isolamento social, vulnerabilidade socioeconômica e barreiras de acesso aos serviços de saúde, permitem orientar políticas públicas e programas de prevenção mais contextualizados. A predominância de métodos quantitativos e questionários domiciliares demonstra a viabilidade de monitorar a violência em áreas rurais, apontando caminhos para o fortalecimento da vigilância e coleta de dados. Também se evidenciaram lacunas metodológicas e a escassez de estudos nacionais, reforçando a necessidade de novas pesquisas que considerem as especificidades do rural. Por fim, a caracterização das variáveis sociodemográficas, econômicas e culturais e a exposição das populações vulneráveis fornecem subsídios para ações de saúde e educação sensíveis ao contexto, além de fortalecer iniciativas de conscientização, contribuindo para a prevenção e mitigação da violência neste cenário. Como limitações, ressalta-se a não recuperação de alguns registros, majoritariamente com mais de 10 anos de publicação, e o fato de que alguns estudos não descreveram as técnicas de coleta de dados.

### Avanços Para a Área da Saúde e Enfermagem

Para a saúde, em especial a enfermagem, o mapeamento dos métodos empregados em inquéritos populacionais sobre as violências em áreas rurais evidencia particularidades e desafios desses contextos. A criação e adaptação de instrumentos de coleta de dados específicos para a realidade rural garantem informações mais precisas, favorecem a identificação de subgrupos populacionais mais vulneráveis e subsidiam a avaliação de programas de prevenção e enfrentamento da violência. Além disso, tais achados fortalecem a base de evidências científicas e orientam intervenções mais eficazes. Ao envolver as comunidades rurais na pesquisa, os enfermeiros promovem empoderamento e participação social, fomentando soluções conjuntas para o problema da violência.

## CONCLUSÃO

O mapeamento dos inquéritos populacionais que abordam as violências contra pessoas residentes em áreas rurais revelou uma lacuna significativa de estudos com metodologias robustas e padronizadas. Os estudos identificados apresentaram grande heterogeneidade quanto aos instrumentos utilizados, tamanhos amostrais e delineamentos metodológicos. Além disso, a complexidade do rural, associada à dificuldade de acesso e à escassez de recursos, representa um desafio para a realização de pesquisas nessa área.

## DISPONIBILIDADE DE DADOS

Todo o conjunto de dados que dá suporte aos resultados deste estudo está disponível mediante solicitação ao autor correspondente.

# Translation: Metodologías de encuestas poblacionales sobre la violencia en zonas rurales: revisión del alcance

**DOI:** 10.1590/1980-220X-REEUSP-2025-0239es

**Authors:** Carmem Layana Bandeira Bervig, Marta Cocco, Jaqueline Arboit, Ethel Bastos da Silva, Cristiane Cardoso de Paula, Manuel Gaspar da Silva Lisboa

**Affiliations:** 1 Universidade Federal do Rio Grande do Sul, Escola de Enfermagem, Porto Alegre, RS, Brasil.; 2 Universidade Federal de Santa Maria, Departamento de Ciências da Saúde, Palmeira das Missões, RS, Brasil.; 3 Universidade Federal de Santa Maria, Centro de Ciências da Saúde, Departamento de Enfermagem, Santa Maria, RS, Brasil.; 4 Universidade Nova de Lisboa, Faculdade de Ciências Sociais e Humanas, Departamento de Sociologia, Lisboa, Portugal.

**Keywords:** Violência, Salud Rural, Encuestas y Cuestionarios, Revisión de Alcance

## Abstract

**Objetivo:**

Examinar los métodos empleados en las encuestas poblacionales sobre la epidemiología de la violencia contra las personas que viven en zonas rurales.

**Método:**

Revisión de alcance, realizada según el método JBI y reportada según PRISMA-ScR. El protocolo se registró en OSF en agosto de 2022 (DOI https://doi.org/10.17605/OSF.IO/WG6PU). Las búsquedas se realizaron de julio a septiembre de 2022, con una actualización el 27 de abril de 2025, mediante el mapeo de palabras clave y términos controlados, guiado por el acrónimo Población, Concepto y Contexto. Las fuentes de datos incluyeron MEDLINE, Web of Science, LILACS, Scopus, EMBASE, CINAHL, Cochrane Library, literatura gris y estudios no publicados. Dos revisores independientes realizaron la selección de los estudios.

**Resultados:**

Se incluyeron 110 estudios: el 89.09% (n = 98) con diseño transversal; el 46.36% (n = 51) con adultos; y el 56.36% (n = 62) con mujeres. En el 37,27% (n = 41) de los estudios no se describió la técnica de muestreo; el 59,09% (n = 65) utilizó cuestionarios; y el 69,09% (n = 76) realizó análisis estadísticos descriptivos e inferenciales.

**Conclusión:**

Se observó heterogeneidad en cuanto a los instrumentos, el muestreo y los diseños metodológicos de las encuestas.

## INTRODUCCIÓN

La violencia constituye un grave problema de salud global, que exige esfuerzos intersectoriales y recursos individuales e institucionales, a menudo limitados para su enfrentamiento efectivo^(1)^. Estadísticamente, se trata de un fenómeno difícil de medir, dada su complejidad, invisibilidad social y subnotificación^(1,2)^. La Organización Mundial de la Salud define la violencia como el uso intencional de la fuerza o el poder, real o amenazado, contra uno mismo o contra otros, que resulta o puede resultar en lesiones, muerte, daño psicológico, discapacidad del desarrollo o privación^(3)^.

En el contexto rural, la violencia adquiere contornos aún más complejos debido a la distancia geográfica con respecto a las ciudades, la falta de transporte público, las barreras de acceso a los servicios de salud, la precariedad de la infraestructura y la falta de insumos^(4)^. Las personas que residen en estas zonas se enfrentan a múltiples formas de violencia —física, psicológica, sexual y económica— perpetradas por familiares, compañeros, miembros de la comunidad y, en ocasiones, profesionales de la salud^(3-7)^. Aunque también ocurre en las zonas urbanas, la subnotificación es particularmente elevada en el medio rural, debido a las dificultades de acceso a los servicios de protección y a los factores socioculturales que naturalizan la violencia^(1)^. Estos obstáculos aumentan la vulnerabilidad de la población rural, reforzando la invisibilidad del fenómeno^(4)^.

Las encuestas de población son instrumentos esenciales para la medición epidemiológica de la violencia, ya que permiten estimar prevalencias, mapear factores asociados y subsidiar políticas públicas de prevención y enfrentamiento. Estos estudios ofrecen información sobre los tipos de violencia, los factores de riesgo, las consecuencias y los impactos en diferentes contextos sociales, y son fundamentales para comprender la dimensión del problema^(8,9)^. Importantes encuestas nacionales, como la Encuesta Nacional de Salud (ENS)^(9)^, incluyen las zonas rurales en sus muestras; sin embargo, rara vez exploran en profundidad los resultados en materia de salud y las particularidades de la recolección de datos en contextos rurales en lo que respecta a la violencia.

A pesar de la relevancia de las encuestas, faltan sistematizaciones que examinen de manera exhaustiva los métodos empleados en las encuestas poblacionales destinadas a medir la violencia en las zonas rurales. Esta laguna compromete la comparabilidad de los datos, limita el fortalecimiento de la base empírica y dificulta la planificación de acciones intersectoriales y el seguimiento de las políticas de equidad. Los estudios de base poblacional siguen siendo dispersos, metodológicamente heterogéneos y, a menudo, inadecuados para captar las singularidades territoriales, culturales y de acceso de la población que reside fuera de los centros urbanos. Por lo tanto, las encuestas poblacionales son fundamentales para suplir esta carencia, ya que recopilan información directamente de los residentes, abordan diferentes formas de violencia^(9,10)^y revelan patrones de violencia que a menudo se descuidan en las políticas públicas y en la asignación de recursos para la prevención y mitigación del fenómeno^(8,9)^.

En vista de lo anterior, este estudio tiene como objetivo orientar la elección de los métodos más adecuados para la realización de encuestas poblacionales sobre la violencia en las zonas rurales, centrándose en técnicas de recolección y análisis de datos que tengan en cuenta variables sociodemográficas, culturales, económicas y otros marcadores sociales. De este modo, esta revisión de alcance tuvo como objetivo examinar los métodos empleados en las encuestas poblacionales relativas a la epidemiología de la violencia contra las personas residentes en zonas rurales.

## MÉTODO

### Tipo de Estudio

Se trata de una revisión de alcance realizada de acuerdo con el método del JBI y descrita según las directrices de los Elementos de información preferidos para revisiones sistemáticas y metaanálisis, extensión para revisiones de alcance (PRISMA-ScR)^(11,12)^. El JBI es una institución internacional dedicada a la síntesis, transferencia e implementación de evidencia en salud, reconocida por el rigor y la actualización de sus diseños para revisiones sistemáticas y de alcance. Por lo tanto, la elección de este marco de referencia se justifica por la solidez metodológica que confiere a la revisión^(11)^. Cabe destacar que esta revisión se centra en las estrategias metodológicas empleadas en las encuestas poblacionales, sin entrar en la categorización de los tipos de violencia abordados, ya que se trata de un eje analítico distinto.

Para su desarrollo, se siguieron las siguientes etapas: definición del objetivo, la pregunta de revisión y los criterios de selección; planificación y ejecución de la estrategia de búsqueda; selección y extracción de datos; análisis y presentación de datos; y resumen de las evidencias mapeadas de acuerdo con el objetivo de la revisión^(11)^.

Inicialmente se consultaron*Open Science Framework*(OSF),*Medical Literature Analysis and Retrieval System Online* (MEDLINE),*Cochrane Database of Systematic Reviews*, PROSPERO (*International prospective register of systematic reviews*) y JBI*Evidence Synthesis*, sin que se identificaran revisiones de alcance publicadas o en curso sobre encuestas poblacionales relativas a la epidemiología de la violencia en el contexto rural. Ante esto, se elaboró el protocolo de revisión y se registró públicamente en el OSF el 20 de agosto de 2022 (DOI https://doi.org/10.17605/OSF.IO/WG6PU).

### Identificación de la Cuestión de Revisión

La cuestión de revisión se estructuró basándose en el acrónimo PCC (Población, Concepto y Contexto)^(11)^, siendo: P: personas en situación de violencia; C: métodos de encuestas de base poblacional; y C: áreas rurales. A partir de esta combinación mnemónica, se formuló la pregunta: ¿Qué métodos se emplean en las encuestas poblacionales relativas a la epidemiología de la violencia contra las personas que residen en zonas rurales?

### Criterios de Selección

Los criterios de inclusión y exclusión se definieron de acuerdo con los elementos del acrónimo PCC. Población (P): estudios con niños, adolescentes, adultos y personas mayores en situación de violencia, sin ninguna restricción en cuanto a religión, orientación sexual, identidad de género, discapacidad u otros marcadores sociales. Se adoptaron los siguientes grupos de edad: niños (hasta 12 años incompletos); adolescentes (12 años completos hasta 18 años incompletos); adultos (19 años completos hasta 59 años); y ancianos (60 años o más)^(5)^. Se excluyeron los estudios centrados en las consecuencias de la violencia para la salud, ya que abordaban resultados clínicos y no aspectos metodológicos, lo que no se corresponde con el objetivo de esta revisión. Concepto (C): estudios con métodos de encuestas poblacionales. Contexto (C): estudios que abordaban el área rural. Se excluyeron los estudios que analizaban datos de áreas urbanas y rurales de forma conjunta.

Se incluyeron artículos de estudios experimentales y cuasi experimentales (ensayos clínicos controlados aleatorios y no aleatorios; estudios del tipo antes y después; series temporales); estudios observacionales analíticos (estudios de cohortes prospectivos y retrospectivos, de casos y controles, transversales analíticos); diseños de investigación documental (como el análisis de expedientes médicos); estudios observacionales descriptivos (series de casos, informes de casos individuales y transversales descriptivos); además de literatura gris (tesis, tesis doctorales, monografías); disponibles en portugués, inglés o español. Se excluyeron los estudios cualitativos, de métodos mixtos, de creación o validación de instrumentos, estudios de intervención, notas previas, estudios de revisión, protocolos de investigación y editoriales. No se aplicaron restricciones en cuanto al período de publicación.

### Estrategias de Búsqueda

La JBI recomienda utilizar una estrategia de búsqueda en tres etapas, con el objetivo de recuperar estudios elegibles, publicados y no publicados^(11)^. Las búsquedas bibliográficas se realizaron con la colaboración de una bibliotecaria especializada en la recuperación de información en salud y certificada por el JBI, que llevó a cabo la cartografía de las palabras clave y los términos estandarizados en los vocabularios controlados: Descriptores en Ciencias de la Salud (DECS), a través del Portal Regional de la Biblioteca Virtual en Salud (BVS); Medical Subject Healding (MESH) a través de Pubmed; y Emtree (Embase subject headings), de la base de datos EMBASE (Elsevier). En la primera etapa, se realizó una búsqueda inicial combinando palabras clave y descriptores en los títulos de temas médicos en MEDLINE a través de PubMed y en el Portal de la BVS, para mapear los términos relevantes. A continuación, se procedió al análisis de los títulos y resúmenes de los artículos recuperados, con el fin de recopilar palabras clave y términos de índice comúnmente utilizados.

En la segunda etapa, se incluyeron términos adicionales extraídos de los títulos, resúmenes y descriptores (MeSH/DeCS/Emtree) de los artículos, que se aplicaron en los campos título, resumen y tema (Subject Headings, Mesh Terms, Emtree Terms, Keywords), lo que permitió formular una estrategia de búsqueda final, aplicada en PubMed en septiembre de 2022 (Cuadro 1). Esta estrategia se adaptó posteriormente al resto de fuentes de información incluidas. Las estrategias completas de todas las fuentes y los archivos de extracción están disponibles públicamente en*SciELO Data* (https://doi.org/10.48331/SCIELODATA.MU1AB9), lo que garantiza la transparencia y la reproducibilidad. En la tercera etapa, se examinaron las listas de referencias de los estudios incluidos de acuerdo con los criterios de elegibilidad, con el fin de localizar otros estudios potencialmente relevantes para la revisión.

**Cuadro 1:** Estrategia de búsqueda en Pubmed aplicada el 23 de septiembre de 2022 – Palmeira das Missões, RS, Brasil, 2023.

Búsqueda	Estrategia de búsqueda	Resultados
#1	Search: “Violence”[mh] violence*[tiab] OR Crime*[tiab] OR Offense*[tiab] OR Abuse*[tiab] OR “Aggression”[mh] OR Aggression*[tiab] OR “Homicide”[mh] OR Homicide*[tiab] OR femicide*[tiab] OR mistreatment[tiab] Sort by: Most Recent	273,117
#2	Search: “Surveys and Questionnaires”[mh] OR Survey*[tiab] OR Nonrespondent*[tiab] OR Questionnaire*[tiab] OR Respondent*[tiab] OR “Health Surveys”[mh] OR “Health Survey”[tiab] OR “National Health Survey”[tiab] OR Prevalence[mh] OR Prevalence*[tiab] OR Incidence*[tiab] OR Epidemiology[mh] OR Epidemiolog*[tiab] OR “Health Surveys”[mh] OR Health Survey*[tiab] OR “population-based survey”[tiab] OR “Epidemiologic Studies”[mh] OR “Epidemiological Studies”[tiab] OR “Epidemiological Study”[tiab] OR “Epidemiologic Study”[tiab] OR “population-based household survey”[tiab] OR “population-based survey”[tiab] OR “population-based household survey”[tiab] OR “population survey”[tiab] Sort by: Most Recent	5,493,787
#3	Search: Rural Area*[tiab] OR Rural Zone*[tiab] OR “Rural Population”[mh] OR “Medium Communities”[tiab] OR Rural Communit*[tiab] OR Rural Population*[tiab] OR Rural Settlement*[tiab] OR Rural Spatial Distribution*[tiab] OR Small Communit*[tiab] OR “peasant farmers”[tiab] OR peasant[tiab] OR cottager*[tiab] OR Rural[tiab] OR countryside[tiab] OR “agricultural area”[tiab] OR “rural environment”[tiab] OR “rural land”[tiab] OR “rural dweller”[tiab] OR “rural people”[tiab] OR “rural resident”[tiab] OR “rural society”[tiab] OR “rural worker”[tiab] OR “village people”[tiab] OR “village population”[tiab] OR “village resident”[tiab] OR villager[tiab] Sort by: Most Recent	183,615
#4	Search: #1 AND #2 AND #3 Sort by: Most Recent	2,527

Fuente: Pubmed, (Org) autora.

La s fuentes de datos consultadas incluyeron: Medical Literature Analysis and Retrieval System Online (MEDLINE/PubMed), Web of Science Core Collection/Clarivate Analytics, Literatura Latinoamericana y del Caribe en Ciencias de la Salud (LILACS/BVS), SciVerse Scopus/Elsevier, EMBASE/Elsevier, Índice acumulativo de enfermería y salud aliada (CINAHL/EBSCO), Biblioteca Cochrane/Wiley. Las fuentes de estudios no publicados y literatura gris incluyeron: DART-E, EthoS, Tesis y Disertaciones de Acceso Abierto (OATD), Open Grey, Biblioteca Digital en Red de Tesis y Disertaciones (NDLTD), ProQuest Dissertations & Theses Global (PQDT) y Banco de Tesis de la Coordinación de Perfeccionamiento del Personal de Nivel Superior (CAPES). La búsqueda manual de referencias se realizó a través de Google Scholar. Las búsquedas bibliográficas se llevaron a cabo entre julio y septiembre de 2022 y se actualizaron el 27 de abril de 2025, volviendo a aplicar la estrategia adaptada a cada base.

### Selección de las Fuentes de Evidencia

Tras la búsqueda de estudios en las fuentes de datos, todos los registros identificados se exportaron al gestor de referencias Endnote y, tras eliminar los duplicados, se importaron al software Rayyan. A continuación, dos revisores independientes realizaron la selección mediante la lectura de títulos/resúmenes en modo ciego (Blind ON/OFF). En los casos en que hubo divergencias, se contactó a un tercer revisor.

La selección fue precedida por una prueba piloto de 25 títulos/resúmenes por revisor. Tras la discusión del equipo y alcanzado un consenso del 75%, se continuó con la selección^(13)^. Para garantizar la fiabilidad del proceso, se desarrolló una lista de verificación con criterios de elegibilidad. Los conflictos entre las revisoras fueron resueltos por la tercera evaluadora y, cuando fue necesario, se contactó por correo electrónico a los autores de los estudios primarios para obtener aclaraciones. En la segunda etapa, se leyeron íntegramente los estudios preseleccionados. Todo el proceso de selección se documentó en un diagrama de flujo, de acuerdo con las directrices de PRISMA-ScR^(12)^.

### Extracción de Datos

Los datos fueron extraídos de los artículos incluidos por dos revisores, de forma independiente, y, en caso de desacuerdo, se consultó a un tercer revisor. Se utilizó un formulario estructurado^(11)^en Microsoft Excel®, previamente probado para verificar su adecuación a la pregunta de revisión. Los revisores fueron debidamente capacitados para llevar a cabo esta etapa. La información extraída abarcó: autor(es), año de publicación, país de origen, objetivo, población y muestra, diseño del estudio, técnicas de recopilación y análisis de datos, así como variables sociodemográficas relacionadas con las políticas de equidad: discapacidad, orientación sexual, identidad de género y otros marcadores sociales de diferencia. La variable violencia en zonas rurales se identificó según la definición adoptada en cada estudio, teniendo en cuenta criterios como la autodeclaración de residencia rural, la clasificación administrativa del territorio o la estratificación geográfica utilizada por los autores originales.

### Análisis y Tratamiento de los Datos

Las pruebas recopiladas se analizaron de forma descriptiva y comparativa, y se presentaron en tablas y gráficos. Los resultados van acompañados de un resumen narrativo que relaciona los hallazgos con el objetivo y la pregunta de la revisión.

### Aspectos Éticos

Al tratarse de una revisión bibliográfica, sin implicación directa de seres humanos y que analizó exclusivamente datos secundarios disponibles públicamente, este estudio no requirió la aprobación de un Comité de Ética en Investigación. Sin embargo, el protocolo fue evaluado y registrado de acuerdo con las recomendaciones internacionales de buenas prácticas en revisiones sistemáticas y de alcance. Cabe destacar que se respetaron los derechos de autor y la integridad de las investigaciones analizadas.

## RESULTADOS

La búsqueda dio como resultado la identificación de 23 717 estudios. Tras eliminar 11 119 duplicados y 584 registros no elegibles, se sometieron 12 014 estudios a una selección por título y resumen. De estos, 11 833 fueron excluidos por no responder a la pregunta de la revisión, quedando 181 para su lectura completa. A partir del análisis de las listas de referencias, se incluyeron tres estudios adicionales. En la lectura completa, 22 no se recuperaron y 49 se excluyeron por no cumplir los criterios de inclusión. Así, 110 estudios compusieron el corpus analítico de la revisión, como se muestra en el diagrama de flujo PRISMA (Figura 1).

**Figura 1:** Diagrama de flujo PRISMA 2020 para nuevas revisiones sistemáticas, incluyendo búsquedas en bases de datos y registros – Palmeira das Misiones/RS, Brasil, 2025.

Leyenda: *BVS (n = 542), Cochrane (n = 347), EBSCO – CINAHL y SocINDEX (n = 3601), Embase (n = 5075), ETHOS (n = 10), POPLINE (n = 99), PUBMED (n = 3089), PsycINFO (n = 200), Scopus (n = 6289), WOS (n = 4388). **Retirada antes de la selección: marcada como no elegible. ***Excluida por no responder a la pregunta de la revisión. ****Razón 1: Población Razón 2: Concepto Razón 3: Contexto.

La distribución geográfica de los estudios incluidos se presenta en la Figura 2, en una escala de 0 a 20. El mapa se elaboró en la plataforma*Infogram*(versión gratuita). Cuanto más intenso es el color, mayor es el número de estudios por país. Se observa un predominio en India (n = 20), Bangladesh (n = 15), Estados Unidos (n = 12) y China (n = 11).

**Figura 2:** Distribución geográfica de los estudios incluidos en la revisión – Palmeira das Missões/RS, 2025.

La Figura 3 muestra la distribución temporal de los estudios, publicados entre 1997 y 2025, con mayor concentración en el periodo comprendido entre 2017 y 2020. Entre 2020 y 2022, años marcados por la pandemia de COVID-19, se identificaron 28 estudios.

**Figura 3:** Número de estudios por año de publicación – Palmeira das Missões/RS, 2025.

Todos los estudios adoptaron un enfoque cuantitativo. De ellos, 98 (89,09%) utilizaron un diseño transversal; seis (5,45%) eran estudios de cohortes; tres (2,73%), series de casos; uno (0,91%), caso-control; uno (0,91%), encuesta de base poblacional; y uno (0,91%), encuesta domiciliaria (Cuadro 2).

**Cuadro 2:** Cuadro con identificación de los estudios – Palmeira das Missões, RS, Brasil, 2025^(7,14–122).^

Año Ref. Referencia	Diseño. número de participantes. rango de edad. sexo	Técnica de muestreo	Técnica de recopilación de datos	Lugar de recopilación de datos	Análisis de datos
2021^(7)^	Transversal. 1681 niños y niñas	Conglomerados en varias etapas	Entrevista	Escuela	Estadística inferencial
1997^(14)^	Transversal. 242 mujeres adultas	No se describe	Cuestionario	No se describe	Estadística descriptiva
1998^(15)^	Transversal. 1693 mujeres adultas	No describe	Cuestionario	Centro de salud	Estadística descriptiva e inferencial
1998^(16)^	Transversal. 155 mujeres adultas	Conveniencia	Formulario de extracción	En lugar privado	Estadística descriptiva e inferencial
2000^(17)^	Transversal. 4643 adolescentes de ambos sexos	Aleatoria sistemática	Entrevista	No describe	Estadística descriptiva e inferencial
2001^(18)^	Serie de casos. 110 documentos de niños y niñas	Aleatoria	No describe	Base de datos	Estadística descriptiva
2003^(19)^	Cohorte. 4904 mujeres adultas	No describe	Entrevista	Domicilio	Estadística descriptiva e inferencial
2003^(20)^	Transversal. 190 mujeres adultas	No describe	Cuestionario	No describe	Estadística inferencial
2004^(21)^	Transversal. 157 niños y adolescentes de ambos sexos	No describe	Cuestionario	Escuela	Estadística descriptiva
2004^(22)^	Transversal. 192 niños y niñas	Conveniencia	Cuestionario	Escuela	Estadística descriptiva
2004^(23)^	Transversal. 500 mujeres adultas	Aleatoria	Entrevista	Domicilio	Estadística descriptiva
2005^(24)^	Transversal. 431 adolescentes y adultas	No describe	No describe	No describe	No se describe
2006^(25)^	Transversal. 512 adolescentes, adultos y ancianos de ambos sexos	Aleatoria	Entrevista	Domicilio	Estadística descriptiva e inferencial
2007^(26)^	Transversal. 20 274 adolescentes de ambos sexos	No describe	Entrevista	Escuela	Estadística inferencial
2007^(27)^	Transversal. 1266 mujeres adultas	No se describe	Cuestionario	Lugar de trabajo	Estadística descriptiva e inferencial
2007^(28)^	Transversal. 3664 mujeres adultas	No se describe	Cuestionario	Centro de salud	Estadística descriptiva e inferencial
2007^(29)^	Transversal. 1078 personas mayores de ambos sexos	Aleatorio	Cuestionario y entrevista	Domicilio	Estadística descriptiva e inferencial
2008^(30)^	Transversal. 883 mujeres adultas	Aleatoria por *conglomerados*	Cuestionario	Domicilio	Estadística descriptiva e inferencial
2008^(31)^	Transversal. 1684 adolescentes de ambos sexos	Aleatoria	Cuestionario	Escuela	Estadística descriptiva e inferencial
2009^(32)^	Transversal y descriptivo. 340 mujeres adultas	Varias etapas	Cuestionario	Domicilio	Estadística descriptiva e inferencial
2009^(33)^	Transversal. 387 mujeres adultas	No describe	Cuestionario	Centro de salud	Estadística descriptiva e inferencial
2009^(34)^	Transversal. 4411 adolescentes y adultas	Aleatoria	Cuestionario y entrevista	Domicilio	Estadística descriptiva e inferencial
2010^(35)^	Transversal. 141 adolescentes y adultas	Aleatoria sistemática	Entrevista	Domicilio	Estadística descriptiva
2010^(36)^	Transversal. 5106 adolescentes de sexo femenino	Aleatoria por conglomerados en dos etapas	Entrevista	Domicilio	Estadística descriptiva e inferencial
2011^(37)^	Transversal. 1502 Adultos y ancianos de sexo femenino	Aleatoria sistemática	Cuestionario y entrevista	Domicilio	Estadística descriptiva
2011^(38)^	Transversal. 1051 personas mayores de ambos sexos	No se describe	Cuestionario	Domicilio	Estadística descriptiva e inferencial
2011^(39)^	Transversal. 765 mujeres adultas	No se describe	Cuestionario	No se describe	Estadística descriptiva e inferencial
2011^(40)^	Transversal. 7432 mujeres adultas	No describe	Cuestionario	No se describe	Estadística descriptiva e inferencial
2011^(41)^	Transversal. 1296 adolescentes y adultos de ambos sexos	Aleatoria sistemática	Cuestionario	Domicilio	Estadística descriptiva e inferencial
2012^(42)^	Encuesta basada en la población. 597 mujeres adultas	Aleatorio	Cuestionario	Domicilio	Estadística descriptiva e inferencial
2012^(43)^	Transversal. 236 personas mayores de ambos sexos	Conveniencia	Entrevista	Domicilio	No se describe
2012^(44)^	Transversal. 2000 personas mayores de ambos sexos	*Cúmulo* en dos etapas	Cuestionario y entrevista	Domicilio	Estadística descriptiva e inferencial
2012^(45)^	Transversal. 1296 adolescentes y adultas	Aleatoria sistemática	Cuestionario	Domicilio	Estadística descriptiva e inferencial
2013^(46)^	Transversal. 235 parejas de sexo masculino y femenino	Conglomerados	Cuestionario	Escuela	Estadística descriptiva e inferencial
2013^(47)^	Transversal. 300 adolescentes y adultas de sexo femenino	Aleatoria simple	Cuestionario	Lugar privado	Estadística descriptiva e inferencial
2013^(48)^	Transversal. 713 casos de niños y niñas	No se describe	Formulario de extracción	Base de datos	Estadística descriptiva
2014^(49)^	Transversal. 1273 mujeres adultas	No se describe	Entrevista	Lugar privado	Estadística descriptiva e inferencial
2014^(50)^	Transversal. 2869 adolescentes y adultos de ambos sexos	Aleatoria	Cuestionario	Domicilio	Estadística descriptiva e inferencial
2014^(51)^	Transversal. 392 adolescentes y adultas	Varias etapas	Cuestionario	Lugar de preferencia	Estadística descriptiva e inferencial
2014^(52)^	Transversal. 345 mujeres adultas	Aleatorio	Cuestionario y entrevista	Lugar privado	Estadística descriptiva e inferencial
2014^(53)^	Transversal. 380 mujeres adultas	Aleatorio	Cuestionario y entrevista	Domicilio	Estadística descriptiva
2014^(54)^	Transversal. 897 personas mayores de ambos sexos	Dos etapas	Cuestionario	Domicilio	Estadística descriptiva e inferencial
2015^(55)^	Transversal y descriptivo. 314 mujeres adultas	No describe	Cuestionario	No describe	Estadística descriptiva e inferencial
2015^(56)^	Cohorte. 973 niños, adolescentes y adultos de ambos sexos	No describe	Entrevista	Centro de salud	Estadística descriptiva
2015^(57)^	Transversal. 220 mujeres adultas	No describe	Cuestionario	No describe	Estadística descriptiva e inferencial
2015^(58)^	Transversal. 493 niños y adolescentes de ambos sexos	Varias etapas	Cuestionario	Escuela	Estadística inferencial
2015^(59)^	Transversal. 4131 niños y adolescentes de ambos sexos	Conglomerados en varias etapas	Cuestionario	Escuela	Estadística descriptiva e inferencial
2016^(60)^	Transversal. 310 mujeres adultas	No describe	Cuestionario	Domicilio	Estadística descriptiva e inferencial
2016^(61)^	Transversal. 694 incidentes de mujeres adultas	No describe	No describe	No describe	No describe
2016^(62)^	Transversal. 355 mujeres adultas	Aleatoria sistemática	Cuestionario	Lugar de preferencia	Estadística descriptiva e inferencial
2017^(63)^	Cohorte. 455 mujeres adultas	No se describe	Examen celular y cuestionario	No se describe	Estadística descriptiva
2017^(64)^	Transversal. 76 mujeres adultas	Varias etapas	Entrevista	No describe	Estadística descriptiva e inferencial
2017^(65)^	Serie de casos. 471 denuncias policiales de violencia contra mujeres adultas	No describe	Formulario de extracción	Base de datos	Estadística descriptiva y espacial
2018^(66)^	Transversal. 200 personas mayores de ambos sexos	No describe	Cuestionario	Lugar Privado	Estadística descriptiva e inferencial
2018^(67)^	Cohorte. 455 mujeres adultas	No se describe	Cuestionario	No se describe	Estadística descriptiva e inferencial
2018^(68)^	Estudio de casos y controles. 214 mujeres adultas	No se describe	Cuestionario	Establecimiento de salud	Estadística descriptiva e inferencial
2018^(69)^	Transversal. 2381 adolescentes y adultas	Aleatoria en varias etapas	Cuestionario y entrevista	Domicilio	Estadística descriptiva e inferencial
2018^(70)^	Serie de casos. 471 denuncias policiales de niños, adolescentes, adultos y ancianos, de sexo masculino y femenino	Conveniencia	Formulario de extracción	Base de datos	No describe
2018^(71)^	Cohorte. 307 niños y niñas	No describe	Cuestionario	Escuela	Estadística inferencial
2018^(72)^	Transversal. 1484 adolescentes y adultos de ambos sexos	Varias etapas	Cuestionario y entrevista	Domicilio	Estadística descriptiva e inferencial
2018^(73)^	Transversal. 800 adolescentes y adultas	Aleatoria	Entrevista	Lugar privado	Estadística descriptiva e inferencial
2018^(74)^	Transversal. 339 personas mayores de ambos sexos	Aleatoria	Entrevista	No describe	Estadística descriptiva e inferencial
2019^(75)^	Transversal. 409 mujeres adultas	Consecutiva	Cuestionario	Domicilio	Estadística descriptiva e inferencial
2019^(76)^	Transversal. 409 adolescentes y adultas	No se describe	Cuestionario	Establecimiento de salud	Estadística descriptiva e inferencial
2019^(77)^	Transversal. 337 adultos de sexo femenino y masculino	No describe	Entrevista	Domicilio	Estadística descriptiva e inferencial
2019^(78)^	Transversal. 442 adolescentes de ambos sexos	No se describe	Entrevista	Escuela	Estadística descriptiva e inferencial
2019^(79)^	Transversal. 1326 niños y niñas	Agrupaciones aleatorias	Cuestionario	Escuela	Estadística descriptiva e inferencial
2019^(80)^	Transversal. 1320 niños y niñas.	Aleatoria estratificada	Cuestionario	No describe	Estadística inferencial
2019^(81)^	Transversal. 980 mujeres adultas	Aleatoria sistemática	Cuestionario	Domicilio	No se describe
2019^(82)^	Transversal. 301 mujeres adultas	Aleatorio simple	Cuestionario	No se describe	Estadística descriptiva e inferencial
2019^(83)^	Transversal. 243 personas mayores de ambos sexos	No describe	Cuestionario	Domicilio	Estadística descriptiva e inferencial
2019^(84)^	Transversal. 116 mujeres adultas	Aleatoria simple	Cuestionario	Domicilio	No se describe
2019^(85)^	Transversal. 1000 hombres adultos	Aleatorio en varias etapas	Cuestionario	Domicilio	Estadística descriptiva e inferencial
2019^(86)^	Transversal. 1416 niños y niñas	Aleatoria simple	Cuestionario	Lugar Privado	Estadística descriptiva e inferencial
2019^(87)^	Transversal. 137 personas mayores de ambos sexos	No describe	Cuestionario	No describe	No se describe
2020^(88)^	Encuesta domiciliaria. 729 adolescentes de sexo femenino	No describe	Entrevista	Hogar	Estadística descriptiva e inferencial
2020^(89)^	Transversal. 1429 mujeres adultas	Probabilística	Entrevista	Domicilio	Estadística inferencial
2020^(90)^	Transversal. 538 adolescentes y adultas	*Cúmulo* en varias etapas	Cuestionario	Centro de salud	Estadística descriptiva e inferencial
2020^(91)^	Transversal. 3409 niños y cuidadores adultos, de ambos sexos.	Varias etapas	Cuestionario	Domicilio	Estadística descriptiva
2020^(92)^	Transversal. 776 mujeres adultas	Varias etapas	Cuestionario	Domicilio	No se describe
2020^(93)^	Transversal. 246 personas mayores de ambos sexos	Aleatorio	Cuestionario y entrevista	Domicilio	Estadística descriptiva e inferencial
2020^(94)^	Transversal. 213 782 adolescentes de ambos sexos	No describe	No describe	Base de datos	Estadística descriptiva
2020^(95)^	Transversal. 3966 mujeres adultas	No describe	Cuestionario	Domicilio	Estadística descriptiva e inferencial
2020^(96)^	Transversal. 250 adolescentes y adultas	Aleatoria simple	Entrevista	Domicilio	Estadística descriptiva e inferencial
2020^(97)^	Transversal. 5440 mujeres adultas	Aleatoria	Cuestionario	Domicilio	Estadística descriptiva e inferencial
2020^(98)^	Transversal. 5440 mujeres adultas	Aleatoria	Cuestionario	Domicilio	Estadística descriptiva e inferencial
2020^(99)^	Transversal. 152 personas mayores de ambos sexos	Aleatoria simple	Cuestionario y formulario	Centro de salud	Estadística descriptiva e inferencial
2021^(100)^	Transversal. 280 adultos de sexo femenino y masculino	No describe	Cuestionario	No describe	Estadística descriptiva e inferencial
2021^(101)^	Transversal. 677 mujeres adultas	Aleatorio	Cuestionario	Lugar privado	Estadística descriptiva e inferencial
2021^(102)^	Transversal. 373 mujeres adultas	Varias etapas	Cuestionario	Centro de salud	Estadística descriptiva e inferencial
2021^(103)^	Transversal. 655 adolescentes y adultos de ambos sexos	No describe	Cuestionario	Escuela	Estadística descriptiva e inferencial
2021^(104)^	Transversal. 4943 niños y niñas	*Cúmulo* en varias etapas	Cuestionario	Escuela	Estadística descriptiva e inferencial
2021^(105)^	Transversal. 140 mujeres adultas	No describe	Cuestionario	Lugar privado	Estadística descriptiva e inferencial
2021^(106)^	Transversal. 290 mujeres adultas	Aleatoria sistemática	Cuestionario	No describe	Estadística descriptiva e inferencial
2021^(107)^	Transversal. 1929 adolescentes y adultos de ambos sexos	*Cúmulo*	Cuestionario	Domicilio	Estadística descriptiva e inferencial
2022^(108)^	Transversal. 336 mujeres adultas	Probabilística	Cuestionario	Domicilio	Estadística descriptiva e inferencial
2022^(109)^	Transversal. 1062 niños y adolescentes de ambos sexos	Aleatoria	Cuestionario	Domicilio	Estadística descriptiva e inferencial
2022^(110)^	Transversal. 3137 mujeres adultas	No se describe	Cuestionario	No se describe	Estadística descriptiva e inferencial
2022^(111)^	Transversal. 623 mujeres adultas	Aleatoria simple	Entrevista	Domicilio	Estadística descriptiva e inferencial
2022^(112)^	Transversal. 500 adolescentes del sexo femenino	Aleatoria	Entrevista	Escuela	Estadística descriptiva e inferencial
2022^(113)^	Transversal. 945 mujeres adultas	No describe	Cuestionario y entrevista	No se describe	Estadística inferencial
2022^(114)^	Transversal. 44 adolescentes, adultos y ancianos de ambos sexos	No describe	Cuestionario	Hogares	Estadística descriptiva e inferencial
2023^(115)^	Transversal. 238 niños y adolescentes de ambos sexos	Aleatorio	Formulario de extracción	Base de datos	Estadística descriptiva e inferencial
2023^(116)^	Transversal. 450 adultos de ambos sexos	Varias etapas	Cuestionario	Domicilio	Estadística descriptiva e inferencial
2023^(117)^	Transversal. 365 mujeres adultas	Aleatoria	Cuestionario	Domicilio	Estadística descriptiva e inferencial
2024^(118)^	Transversal. 79 229 mujeres adultas	No describe	Formulario de extracción	Base de datos	Estadística descriptiva e inferencial
2024^(119)^	Transversal. 100 niños y niñas	Bola de nieve	Cuestionario	Domicilio	Estadística inferencial
2025^(120)^	Transversal. 100 niños y niñas	Bola de nieve	Cuestionario	Domicilio	Estadística inferencial
2025^(121)^	707 adolescentes, adultas y ancianas	Aleatorio	Entrevista	Domicilio	Estadística inferencial
2025^(122)^	Cohorte. 933 mujeres adultas	Conveniencia	Entrevista	Domicilio	Estadística inferencial

El total de participantes incluidos en los estudios fue de 431 991 individuos (Cuadro 2). La distribución poblacional fue la siguiente: 247 160 adolescentes (57.20%); 131 347 adultos (30.40%); 19 582 adolescentes y adultos (4.53%); 13 424 niños (3.11%); 6,579 personas mayores (1,52%); 6,281 niños y adolescentes (1,45%); 3,409 niños y adultos (0,79%); 1,502 adultos y personas mayores (0,35%); 1,263 adolescentes, adultos y personas mayores (0,29%); 973 niños, adolescentes y adultos (0,23%); y 471 niños, adolescentes, adultos y personas mayores (0,11%). Cabe destacar que la distribución se presentó de esta forma debido a la ausencia de datos estratificados en algunos estudios, que solo indicaron el número total de participantes.

En cuanto al grupo de edad de los participantes, 51 estudios (46.36%) incluyeron adultos; 16 (14.55%), adolescentes y adultos; 11 (10.00%), personas de edad avanzada; 10 (9.09%), niños; ocho (7.27%), adolescentes; siete (6.36%), niños y adolescentes; tres (2,73%), adolescentes, adultos y personas mayores; uno (0,91%), niños y adultos; uno (0,91%), niños, adolescentes y adultos; uno (0,91%), niños, adolescentes, adultos y personas mayores; y uno (0,91%), adultos y personas mayores (Cuadro 2).

En cuanto al sexo de los participantes, 62 (56,36%) estudios se realizaron exclusivamente con personas de sexo femenino; 47 (42,73%), con personas de ambos sexos; y un estudio (0,91%) contó solo con participantes de sexo masculino. Teniendo en cuenta el total de participantes, los estudios que incluyeron a hombres y mujeres sumaron 282 053 individuos (65,29%), mientras que 148 938 (34,49%) eran mujeres y 1000 (0,23%) eran hombres (Cuadro 2).

En relación a la definición de la muestra, 41 (37.27%) estudios no describen la técnica utilizada, 21 (19.09%) adoptaron el muestreo aleatorio y 11 (10.00%), el muestreo en múltiples etapas. Del total de producciones analizadas, 65 (59,09%) emplearon cuestionarios, 23 (20,91%) utilizaron entrevistas y 10 (9,09%) combinaron cuestionarios y entrevistas. La mayoría de las encuestas poblacionales se realizaron en domicilios: 47 (42,73%); 20 (18,18%) no especificaron el lugar de recolección; 14 (12,73%) se desarrollaron en escuelas; y 10 (9,09%), en lugares privados (Cuadro 2).

Para el análisis estadístico, 76 (69,09%) estudios utilizaron estadística descriptiva e inferencial, 13 (11,82%) aplicaron solo estadística descriptiva, 12 (10,91%) emplearon exclusivamente estadística inferencial, ocho (7,27%) no describieron los métodos estadísticos y un estudio (0,91%) utilizó estadística descriptiva y espacial (Cuadro 2).

Las variables sociodemográficas y culturales consideradas en los estudios incluyeron edad, sexo, religión, etnia, nivel educativo, número y sexo de los hijos, estado civil, composición familiar, ingresos, ocupación y características del domicilio (como el número de habitaciones). Las variables relacionadas con las políticas de equidad contemplaron la discapacidad, la orientación sexual, la identidad de género, la generación y otros marcadores sociales de diferencia, además de aspectos directamente relacionados con la violencia, como el historial y el lugar de ocurrencia, el principal agresor, el tipo de violencia y la presencia de discapacidad. Por último, las variables relacionadas con el historial de salud abarcaron las condiciones clínicas, el uso de medicamentos, el historial de enfermedades y los embarazos previos.

## DISCUSIÓN

En la investigación en cuestión, se identificó un perfil de estudios cuantitativos con diseño transversal, observándose un aumento en el número de publicaciones durante el período de la pandemia, así como una mayor incidencia de víctimas de sexo femenino. En cuanto a los participantes, predominaron los adolescentes, mientras que, en el grupo de edad, las mujeres adultas fueron mayoría. El continente asiático concentró el mayor número de publicaciones. En cuanto a la definición de la muestra, la mayoría de los estudios no especificaron la técnica de muestreo utilizada. En la recolección de datos, prevaleció el uso de cuestionarios. Los datos se recopilaron principalmente en los hogares. El análisis estadístico adoptado en los estudios fue, en su mayoría, descriptivo e inferencial.

La opción por el enfoque cuantitativo revela el énfasis de los estudios en examinar las relaciones entre variables como estrategia fundamental para responder a las preguntas e hipótesis formuladas^(69,123)^. Desde esta perspectiva, cuando el objeto de investigación es la violencia, la aplicación de instrumentos cuantitativos en muestras poblacionales permite identificar tendencias estadísticas y establecer correlaciones significativas entre variables^(2,82,101)^.

Los estudios transversales constituyeron el diseño metodológico más utilizado por las investigaciones incluidas en esta revisión. Esta elección se justifica, sobre todo, por el hecho de que, en diversas situaciones, el interés del investigador reside en estimar los parámetros de una población objetivo, verificar la prevalencia y los tipos de violencia más recurrentes, así como establecer un perfil clínico-epidemiológico^(85,97,113,114)^. En este sentido, los estudios transversales buscan delimitar dichos parámetros y formular hipótesis sobre posibles relaciones entre variables dependientes e independientes, considerando medidas puntuales en el tiempo^(115,124)^.

Se observó un aumento en el número de publicaciones sobre el tema en el período 2017–2022. Específicamente en el período de 2020 a 2022, la pandemia de COVID-19 (SARS-CoV-2) provocó múltiples impactos en la salud de la población mundial, incluido el agravamiento de las situaciones de violencia^(112)^. El distanciamiento social, adoptado como estrategia para contener la propagación del virus, contribuyó a la intensificación de la violencia en sus diferentes expresiones, afectando de manera particular a mujeres, personas mayores, niños y adolescentes, en diferentes países y estratos sociales^(8,108)^. En Brasil y en otras partes del mundo, se han registrado aumentos en los índices de violencia contra las mujeres desde la implementación de medidas de aislamiento y confinamiento^(3,100,111)^. Las líneas directas de denuncia indicaron un crecimiento del 84% en Argentina, del 12% en Colombia y del 16% en Perú^(8)^, lo que evidencia un impacto directo en la calidad de vida de las víctimas. En este contexto, el aislamiento, asociado a las condiciones de vulnerabilidad socioeconómica de las mujeres, potenció situaciones de injusticia y exclusión social^(108,109)^.

En el contexto rural, las investigaciones indican que estas medidas han aumentado aún más la fragilidad de la red de apoyo social y el acceso a las estructuras de apoyo, ya limitados antes de la crisis sanitaria^(101,105)^. La suspensión de las actividades colectivas de convivencia y ocio comprometió los espacios de socialización, reduciendo las posibilidades de afrontar y romper el ciclo de violencia^(106,108)^.

Además, la revisión abarcó estudios con niños, adolescentes, adultos y personas mayores^(125-127)^. En cuanto a los adolescentes, que representaron el mayor contingente de participantes en este estudio, las investigaciones revelan que la violencia constituye un reto de salud pública, que provoca alrededor de 227 muertes al día en todo el mundo, además de numerosas hospitalizaciones^(128,129)^. Además, los adolescentes presentan una elevada vulnerabilidad a la violencia física, sexual, psicológica, la negligencia y el acoso escolar^(1,7,79,80,91,97,103,130)^. En Egipto, un estudio reveló que el 15,2% de los adolescentes de zonas rurales habían sido expuestos a violencia física, mientras que el 17,8% había sufrido violencia sexual y el 7,3% había sido víctima de ambos tipos^(88)^. En Brasil, los datos indican que, entre 2016 y 2020, se produjeron 34 918 muertes violentas intencionadas de niños y adolescentes de 0 a 19 años de edad^(1,130)^, lo que refuerza la necesidad de sensibilizar y tomar medidas efectivas frente a este problema^(119,120)^.

Por otro lado, la mayoría de las investigaciones se han realizado con mujeres. En el contexto de la violencia de género, definida como aquella que sufren las mujeres por el hecho de ser mujeres, se estima que aproximadamente una de cada tres mujeres (35%) en el mundo ha sufrido violencia física y/o sexual perpetrada por parejas o terceros a lo largo de su vida^(131)^. Por lo tanto, la violencia de género se configura como un problema global^(3,100,117,118,122)^.

En el ámbito rural, la violencia se ve potenciada por el escenario de adversidades y exclusión que contribuyen a su invisibilidad^(116,118,122,130)^. Las relaciones impregnadas de machismo, autoritarismo y desigualdades de género^(96,111)^, además de la falta de apoyo social, aumentan la vulnerabilidad de las mujeres rurales a la violencia^(6,44,98)^, lo que repercute negativamente en su salud y bienestar^(110,122,132-134)^.

En el continente asiático, donde se realizaron la mayoría de las investigaciones, se destaca la alta prevalencia de violencia contra las mujeres rurales, como en Bangladesh (82,7%)^(132)^y la India (49,5%)^(6)^. Factores como los conflictos étnicos, religiosos y agrarios, la pobreza, las desigualdades y las normas culturales patriarcales contribuyen a la perpetuación de la violencia doméstica y comunitaria^(6,94,96)^.

En Brasil, se observa un número reducido de publicaciones sobre el tema, con solo cinco estudios incluidos en esta revisión. Uno de ellos señala una mayor incidencia de violencia contra las mujeres principalmente los domingos, entre las 12:00 y las 18:00 horas^(65)^. Otro relaciona la violencia con contextos marcados por una elevada vulnerabilidad socioeconómica^(70)^. También hay un estudio que aborda la situación de las personas con discapacidad víctimas de violencia en el medio rural^(114)^, mientras que otro investiga casos de violación de niños y adolescentes en zonas rurales de Alagoas^(115)^. Por último, un estudio presenta una caracterización exhaustiva de las denuncias de violencia contra las mujeres en las zonas rurales de Brasil, en el periodo comprendido entre 2011 y 2020^(118)^.

La aplicación de técnicas de muestreo convencionales en las zonas rurales se ve dificultada por su ubicación remota y de difícil acceso, lo que compromete la representatividad de las muestras^(135)^. La escasa población, los bajos niveles de escolaridad, las altas tasas de analfabetismo, la heterogeneidad de los grupos demográficos y económicos, así como los elevados costos logísticos y de transporte, también limitan el alcance de estas investigaciones^(105,106,135)^. Además, varios estudios no especificaron el método de muestreo adoptado^(136)^, lo que corrobora los hallazgos de esta revisión.

Se observó una diversidad de instrumentos de recolección, sin estandarización entre los estudios, predominando los cuestionarios domiciliarios y las entrevistas estructuradas. Estos instrumentos se emplean ampliamente en las zonas rurales, ya que permiten obtener información objetiva y medible, con relativa practicidad logística^(75,101,102,106–108,137)^. La recolección domiciliaria, predominante en esta revisión, es común en los estudios nacionales^(2)^. Este tipo de recolección permite la atención individualizada y la comprensión del contexto de vida de los participantes^(138)^, aunque puede verse dificultada por el miedo de las víctimas y la presencia del agresor^(94,101,121)^.

En las investigaciones sobre violencia, la elección del análisis estadístico, especialmente el descriptivo y el inferencial, resulta relevante porque permite describir la prevalencia de la violencia en la población, identificar los factores de riesgo asociados y evaluar el impacto de los programas de prevención de la violencia^(99)^. Este enfoque coincide con los hallazgos de la presente revisión.

En cuanto a las variables analizadas, cabe destacar la importancia de considerar la diversidad social, económica y cultural del medio rural^(94,97,98)^. Variables como la edad, el sexo, el nivel educativo, el estado civil y la ocupación son esenciales para caracterizar a esta población^(138)^. Los aspectos económicos y demográficos, como el bajo nivel educativo, los ingresos medios mensuales más bajos y el acceso limitado a los servicios de salud, también son determinantes^(99,113)^. Las cuestiones culturales, como las tradiciones, los valores y las creencias religiosas, influyen directamente en la toma de decisiones y en el nivel de participación en actividades colectivas. En varias zonas rurales prevalecen los lazos entre amigos y vecinos, a partir de actividades de ocio y religiosas, lo que exige sensibilidad cultural a la hora de proponer acciones de salud^(133)^. Además, deben tenerse en cuenta los aspectos relacionados con la orientación sexual, ya que determinados grupos pueden enfrentarse a barreras en cuanto a la aceptación social y el acceso a los servicios de salud^(139)^. Por lo tanto, recopilar datos sobre estas variables en las zonas rurales es fundamental para comprender la diversidad y las características de las poblaciones rurales, lo que permite planificar políticas, programas e intervenciones eficaces para sus necesidades^(138)^.

La violencia en las zonas rurales es un fenómeno amplio y multifacético, que exige comprender las especificidades de su ocurrencia^(3,139)^. Esta revisión contribuye a poner de manifiesto las principales lagunas metodológicas y a estimular la producción de nuevos estudios que tengan en cuenta las especificidades del medio rural. Los resultados refuerzan el papel central de las encuestas de población como instrumentos capaces de captar la complejidad de la violencia en las zonas rurales, señalando vías para mejorar la vigilancia y las políticas públicas de equidad.

### Limitaciones del Estudio

Los hallazgos de esta revisión evidencian características específicas de la violencia en contextos rurales, con posibles contribuciones a la investigación, la política y la práctica en materia de salud. La identificación de grupos vulnerables, como las mujeres, los adolescentes y las personas con discapacidad, así como de factores que intensifican la violencia, como el aislamiento social, la vulnerabilidad socioeconómica y las barreras de acceso a los servicios de salud, permite orientar políticas públicas y programas de prevención más contextualizados. El predominio de métodos cuantitativos y cuestionarios domiciliarios demuestra la viabilidad de monitorear la violencia en las zonas rurales, señalando caminos para fortalecer la vigilancia y la recolección de datos. También se evidenciaron lagunas metodológicas y la escasez de estudios nacionales, lo que refuerza la necesidad de nuevas investigaciones que consideren las especificidades del medio rural. Por último, la caracterización de las variables sociodemográficas, económicas y culturales y la exposición de las poblaciones vulnerables proporcionan insumos para acciones de salud y educación sensibles al contexto, además de fortalecer las iniciativas de concientización, contribuyendo a la prevención y mitigación de la violencia en este escenario. Como limitaciones, cabe destacar la no recuperación de algunos registros, en su mayoría con más de 10 años de publicación, y el hecho de que algunos estudios no describieron las técnicas de recopilación de datos.

### Avances en el Ámbito de la Salud y la Enfermería

En el ámbito de la salud, en especial de la enfermería, el mapeo de los métodos empleados en las encuestas poblacionales sobre la violencia en las zonas rurales pone de manifiesto las particularidades y los retos de estos contextos. La creación y adaptación de instrumentos de recopilación de datos específicos para la realidad rural garantizan una información más precisa, favorecen la identificación de los subgrupos de población más vulnerables y contribuyen a la evaluación de los programas de prevención y lucha contra la violencia. Además, estos hallazgos fortalecen la base de pruebas científicas y orientan intervenciones más eficaces. Al involucrar a las comunidades rurales en la investigación, los enfermeros promueven el empoderamiento y la participación social, fomentando soluciones conjuntas para el problema de la violencia.

## CONCLUSIÓN

El mapeo de las encuestas poblacionales que abordan la violencia contra las personas que residen en zonas rurales reveló una brecha significativa en los estudios con metodologías robustas y estandarizadas. Los estudios identificados presentaron una gran heterogeneidad en cuanto a los instrumentos utilizados, los tamaños de las muestras y los diseños metodológicos. Además, la complejidad del medio rural, asociada a la dificultad de acceso y a la escasez de recursos, representa un reto para la realización de investigaciones en esta área.

## DISPONIBILIDAD DE DATOS

El conjunto de datos que respaldan las conclusiones de este estudio está disponible previa solicitud al autor correspondiente.
